# In Situ Reconstruction of Ag‐Doped Ni_2_P Electrocatalyst for Hydrogen Evolution and Value‐Added Acrylamide Conversion

**DOI:** 10.1002/smll.74237

**Published:** 2026-07-14

**Authors:** Dan Zhang, Zeyi Zhang, Lingshen Meng, Carlos A. Triana, Shangkun Li, Hang Chen, Yiming Li, Greta R. Patzke

**Affiliations:** ^1^ Department of Chemistry University of Zurich Winterthurerstrasse 190 Zurich Switzerland; ^2^ China Frontiers Science Center for Deep Ocean Multispheres and Earth System/Key Laboratory of Marine Chemistry Theory and Technology Ministry of Education Ocean University of China 238 Songling Road Qingdao P. R. China; ^3^ Institute of Photochemistry and Photofunctional Materials University of Shanghai for Science and Technology 516 Jungong Road Shanghai P. R. China

**Keywords:** Ag‐Ni_2_P, acrylamide oxidation, bifunctional catalysts, ethanol, hydrogen evolution reaction

## Abstract

Concurrent remediation of organic pollutants in wastewater and energy‐efficient hydrogen production is a highly attractive yet challenging dual strategy. Acrylamide (AA), depolymerized from polyacrylamide under natural conditions such as UV radiation and elevated temperatures, causes severe environmental pollution. Herein, in situ reconstructed Ag‐doped Ni_2_P nanosheets on Ni foam (CV‐Ag‐Ni_2_P/NF) are developed as a bifunctional catalyst. This catalyst exhibits high activity for the AA oxidation reaction (AOR) while enabling energy‐efficient hydrogen evolution reaction (HER). After electrochemical operation, the initial zipper‐like morphology transforms into spherical features with interspersed rod‐like particles, providing a larger electrochemical surface area and more active sites. Particularly, a potential of 1.82 V delivers 100 mA cm^−2^ in the two‐electrode AOR system. Remarkably, 79.3% AA removal was achieved after 24 h at 50 mA cm^−2^. Meanwhile, ethanol generated at the anode serves as a value‐added product. Density functional theory (DFT) calculations reveal that Ag atoms are the active sites for AA degradation and ethanol formation, with favorable adsorption energies. We propose a plausible AOR catalytic mechanism and reveal the key roles of Ag and Ni in H* adsorption and reduction of AA dehydrogenation energy, which offers a promising way of coupling AOR with HER to reduce energy consumption while producing high‐value products.

## Graphical abstract

We designed a bifunctional electrocatalyst to achieve anodic acrylamide oxidation reaction (AOR) for ethanol production and cathodic hydrogen evolution reaction (HER) to generate high‐value‐added products while reducing energy consumption.

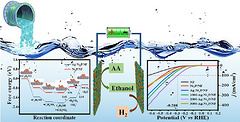

## 1 Introduction

The excessive and persistent use of fossil fuels, including coal, petroleum, and natural gas, has led to extensive environmental pollution, which adds to the growing concerns about global warming [1, 2]. To this end, the utilization of renewable and eco‐friendly sources of energy as a substitute for fossil fuels holds great promise [3, 4, 5]. Hydrogen as an energy carrier, in particular, is considered as a versatile alternative to traditional fossil fuels [6]. Due to its suitability, technological maturity, availability, and convenient large‐scale production, electrochemical water splitting attracts increasing attention and is considered as one of the most efficient strategies [7, 8]. However, the significant electrical energy consumption is the most significant obstacle for the practical use of hydrogen energy from water splitting. The oxygen evolution reaction (OER) suffers from intrinsically slow kinetics, originating from its complex four‐electron transfer mechanism and high activation barriers for dioxygen formation. Due to a thermodynamic equilibrium potential of 1.23 V, large overpotentials are inevitably required at the anode [9, 10]. Replacing the anodic OER with nucleophilic oxidation reactions (NORs) (such as methanol, amine, hydrazine, ethanol, 5‐hydroxymethylfurfural, urea, and glycerol) featuring lower theoretical oxidation potentials can significantly reduce the energy demand of hydrogen production. Importantly, these reactions also offer the possibility of converting organic substrates into value‐added products [11].

Acrylamide (AA), a toxic and highly water‐soluble organic pollutant, is frequently released into aqueous environments from polyacrylamide degradation. Importantly, the AA oxidation reaction (AOR) can serve as an alternative anodic reaction to the sluggish OER, enabling simultaneous pollutant removal and energy‐efficient hydrogen production [12, 13, 14, 15]. Previously, Lv et al. designed a Cu_2_S@Ni_3_Se_2_//Cu_2_S@Ni_3_Se_2_ full‐cell catalyst for urea‐assisted water electrolysis, achieving 10 mA cm^−^^2^ at a cell voltage of 1.48 V. This catalyst also shows high activity for overall water electrolysis, requiring 1.70 V, and outperform the Pt/C‖IrO_2_ benchmark system in urea electrolysis [16]. Thermodynamically favorable primary amine (−CH_2_−NH_2_) electro‐oxidation over a NiSe nanorod array‐based electrode in water has been reported to replace OER for promoting HER. Both H_2_ and value‐added nitriles can be produced at the cathode and anode, while various primary amines can be converted to nitriles with high yields [17]. Similarly, Fei et al. reported a self‐supported nickel phosphide electrode that delivers efficient urea‐assisted hydrogen production at reduced cell voltages. Efficient electrolytic oxidation of organic pollutants provides a promising strategy for energy‐efficient hydrogen production, as well as simultaneous remediation of organic pollutant wastewater, which critically relies on the development of robust and highly active anodic electrocatalysts.

Unlike OER, AOR as well as other NOR are controlled by substrate‐catalyst interactions, where adsorption configuration and bond activation processes dominate the reaction kinetics. Since different fundamental steps require different surface functions, highly efficient NOR electrocatalysts must provide synergistic active sites to achieve organic substrate adsorption, bond breaking, and hydroxyl activation. In transition metal‐based systems, this site synergy typically arises under electrochemical conditions through in situ surface reconstruction [18, 19]. The reconstructed surface can form multi‐active interfaces with altered electronic structures, thereby enhancing control over reaction pathways and selectivity [20]. However, despite the growing interest in NOR, anodic oxidation of acrylamide remains far less explored, particularly with respect to its substrate‐specific reaction mechanisms and corresponding catalyst design principles.

Herein, we introduce an in situ structural reconstruction of Ag‐doped Ni_2_P nanosheets on Ni foam (CV‐Ag‐Ni_2_P/NF) by cyclic voltammetry (CV) treatment, which exhibits excellent electrochemical performance. CV‐Ag‐Ni_2_P/NF behaves as a highly active bifunctional electrocatalyst for generating energy‐efficient H_2_ on the cathode through replacing OER with AOR on the anode, while ethanol was produced simultaneously. CV treatments induce a transformation of the previous zipper‐like morphology into spherical aggregates. The presence of Ag dopants not only provides highly active H* absorption sites, but also lowers the dehydrogenation energy on AOR. By integrating catalyst reconstruction with in situ spectroscopic analysis, this work for the first time provides mechanistic insights into substrate‐specific reaction pathways during acrylamide oxidation which could inspire further research on AOR catalyst design. This bifunctional catalyst shows a high catalytic activity for the AOR accompanied by energy‐efficient electrolytic HER. Furthermore, after 24 h of AOR tests at 50 mA cm^−2^, 79.3% Faraday efficiency of AOR coming along with the production of value‐added ethanol was achieved at the anode. With these findings, we offer a promising route for AA removal from polluted water in a detailed insight which gives a new perspective on transferring the AOR from fundamental research to large‐scale practical application.

## 2 Results and Discussion

### 2.1 Preparation and Characterization of CV‐Ag‐Ni_2_P/NF Electrodes

**FIGURE 1:**
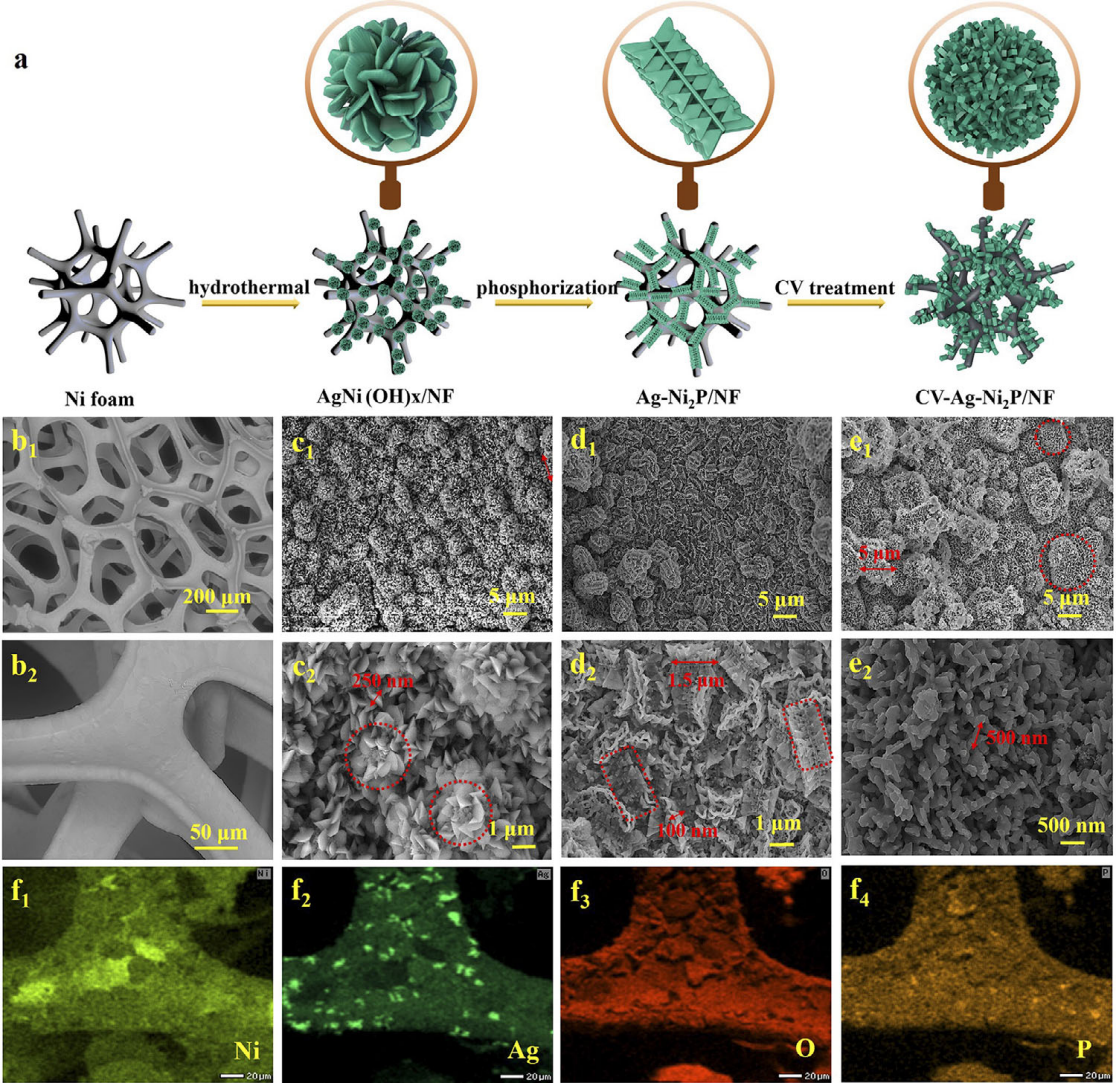
(a) Illustration of CV‐Ag‐Ni_2_P/NF synthesis; SEM images of (b_1‐2_) Ni foam, (c_1‐2_) Ag_0.5_Ni_0.5_(OH)_x_/NF, (d_1‐2_) Ag‐Ni_2_P/NF, (e_1‐2_) CV‐Ag‐Ni_2_P/NF; (f_1‐4_) EDX elemental mapping images of CV‐Ag‐Ni_2_P/NF.

The Ag‐Ni_2_P/NF electrodes were prepared by hydrothermal synthesis using stoichiometric amounts of Ni(NO_3_)_2_·6H_2_O, AgNO_3_, urea and NH_4_F solution together with Ni foam (NF), followed by calcination in Ar atmosphere (cf. Supporting Information for experimental details). The morphology of the electrodes was in situ reconstructed through cyclic voltammetry (CV). The samples prepared with different CV cycles (1000, 2000, and 3000) are referred to as 1000‐CV‐Ag‐Ni_2_P/NF, 2000‐CV‐Ag‐Ni_2_P/NF, and 3000‐CV‐Ag‐Ni_2_P/NF, respectively. The characterizations of the electrodes were performed using multiple analytical techniques, such as SEM, HRTEM, PXRD, XAS, XPS, and Raman spectroscopy (SI for experimental details). The fabrication of the CV‐Ag‐Ni_2_P/NF electrode involves a three‐step synthesis strategy (Figure S1). First, the Ag‐Ni bimetallic hydroxide was formed on the surface of the NF via a hydrothermal approach in presence of Ni(NO_3_)_2_ and AgNO_3_. Second, the resulting Ag_n_Ni_1−n_(OH)_x_/NF was phosphorated to Ag‐Ni_2_P/NF by annealing with NaH_2_PO_2_. As Ag_0.5_Ni_0.5_(OH)_x_/NF electrodes exhibited highest HER, OER, and AOR catalytic performance, they were used in the following sections unless otherwise stated (Figure S2). Finally, the reconstruction of Ag‐Ni_2_P/NF was achieved through CV treatments to attain different structural morphologies (Figure 1a). The commercial NF was used as both the Ni source and conductive substrate.

The microstructure of the as‐prepared samples was characterized by scanning electron microscopy (SEM). The Ni foam displayed a typical 3D framework with a porous structure (Figure 1b_1‐2_). Compared with nickel plate, the larger surface area of Ni foam facilitates the in situ formation of active metal sites. As shown in Figure 1c_1‐2_, Ag_0.5_Ni_0.5_(OH)_x_ formed on NF with a flower‐like nanostructure is arranged in a pattern of nanoflakes surrounding a central site. The nanoflakes had a smooth surface with an average thickness of 250 nm (Figure 1c_2_). Interestingly, the majority of the nanoflakes exhibited growth around a nucleation site, resulting in a flower‐shaped and cluster‐like morphology. The reason for the formation of this morphology might be attributed to the growth of nickel hydroxides around the Ag nanoparticles. After annealing at 320°C in Ar atmosphere, the morphology of the nanoflakes underwent a transformation into a zipper‐like morphology with a width of ∼1.5 µm and a thickness of ∼100 nm (Figure 1d_1‐2_). This can be explained by the uniform arrangement of the previous nanoflake arrays (Ni_2_P) on the Ag nanoparticles, which undergoes a morphological change during annealing, resulting in the formation of a distinctive zipper‐like morphology with high symmetry. After CV treatment the particles on the electrode were transformed into a spherical morphology (diameter: ∼ 5 µm) (Figure 1e_1‐2_). The magnified view (Figure 1e_2_) revealed that the surface of the spherical morphology consists of rod structures with a length of approximate 500 nm. This observation suggests that during the CV treatment, co‐aggregation of the previous zipper‐like morphology (Ag‐Ni_2_P) occurred. It can be clearly seen from Figure S3 that as the number of CV treatment cycles reaches 500, the zipper‐like morphology gradually transforms into spheroid structures. This suggests that most of the Ag nanoparticles aggregate into large particles, thus enabling the simultaneous adsorption of more Ni_2_P on the nanoparticle surface and resulting in the formation of a spherical morphology. Besides, the uniform embedding of CV‐Ag‐Ni_2_P nanoparticles on the surface of NF is evident, which enhances the exposure of reactive sites and increases the surface area of the catalyst. Additionally, the 3D framework structure facilitates the diffusion of H_2_ and electrolyte [21]. Table S1 shows the specific surface area of the electrodes with different morphologies determined by the BET method. After the process of surface phosphidation, the surface area of the catalyst increased to 8.7 m^2^/g because of the heightened number of defects on the NF caused by PH_3_ etching. The BET surface area of the electrodes increased slightly during in situ transformation (from 8.7 to 13.3 m^2^/g), which is in agreement with the transformation of the zipper‐like particle shape into a spherical morphology as revealed by SEM images. As shown in Figure S4 and Figure 1f_1‐4_, Ni, Ag, P, and O were uniformly dispersed on the surface of NF and the shape of elemental maps matched well with it in bright field images, indicating the uniform dispersion of CV‐Ag‐Ni_2_P nanoparticles.

**FIGURE 2:**
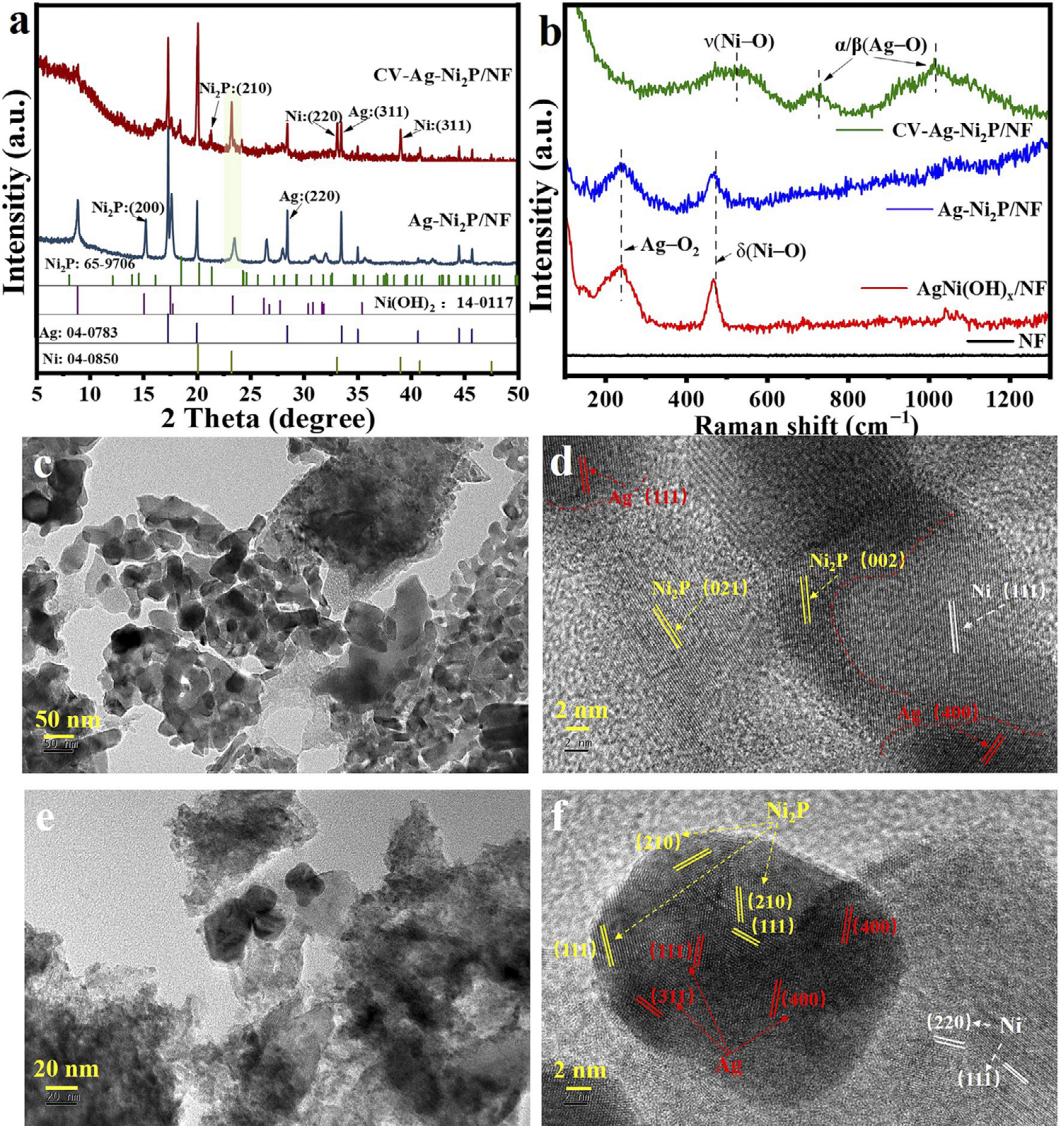
(a) XRD patterns, (b) Raman spectra of the different electrodes, and HRTEM images of (c,d) Ag‐Ni_2_P/NF and (e,f) CV‐Ag‐Ni_2_P/NF.

The structure and phase purity of the phosphide‐based electrodes were analyzed using X‐ray diffraction (XRD) as shown in Figure S5. The diffraction peaks observed at angles of 18.49°, 21.35°, 24.37°, and 32.59° can be attributed to the (111), (210), (002), and (212) crystallographic planes of the Ni_2_P phase (JCPDS No.65‐9706). Meanwhile, the peaks observed at 17.29°, 19.98°, and 28.42° result from the (111), (200), and (220) planes in metallic Ag (JCPDS No.04‐0783). The peaks at 20.08° (111), 23.22° (200), 33.07° (220) and 39.00° (311) are assigned to metallic Ni (JCPDS No.04‐0850) from the Ni‐foam substrate. Compared to the Ag‐Ni_2_P/NF electrode, the exposed crystal planes of CV‐Ag‐Ni_2_P/NF have changed significantly and the crystallinity of the (100) and (200) planes for Ni_2_P has greatly decreased (Figure S5). The exposure of the (210) plane of Ni_2_P, (220) and (311) planes of Ni_,_ and (311) of Ag, indicates that many facets are exposed [22], thereby enhancing electrocatalytic performance (Figure 2a). The changes in chemical bonding are furthermore evident from the Raman spectra of CV‐Ag‐Ni_2_P/NF (Figure 2b). The oxidation peaks at 241 and 472 cm^−1^ are associated with the Ag−O_2_ vibration and bending (δ) Ni−O vibration of NiOOH, respectively. The intensity of these peaks decreased after annealing, demonstrating that the constructed composition on the surface is an Ag−Ni alloy in oxyhydroxides [23]. After CV treatment, it is observed that the band stretching vibration of Ni−O shifts from (δ) Ni−O to (ν) Ni−O. It has been reported that a longer Ni−O bond is typically associated with a higher Raman shift [24]. The 241 cm^−1^ band disappears, while two new bands appear at 602 and 980 cm^−1^, which can be ascribed to the (α) Ag−O and (β) Ag−O modes [25]. After CV treatment, Ag−O_2_ species can transform into (α, β) Ag−O. HRTEM images were collected to check on structural changes. Figure 2c–f shows the TEM images of the samples with or without CV treatment. It is challenging to recognize the actual electrode morphology during HRTEM analysis due to the reconstruction processes and the requirement to peel off the powder from the electrode surface. However, Figure 2c,e clearly reveal the intimate interfacial connection between Ag and Ni_2_P co‐catalysts, especially after the CV treatment. Generally, heterostructures are formed at phase boundaries between the Ni_2_P and Ag phases [26]. It promotes exposure of more active sites and generates an interfacial bonding effect. TEM images also clearly demonstrate that Ni_2_P and Ag nanostructures are highly crystalline, corresponding to the (002) and (021) planes of Ni_2_P, and (111) and (400) planes of Ag, respectively (Figure 2d). After CV treatment, different lattice surfaces of the Ni_2_P ((210), (110), (111)) are exposed and come into contact with the silver lattice surface (Figure 2f). These results agree with the above XRD analyses.

**FIGURE 3:**
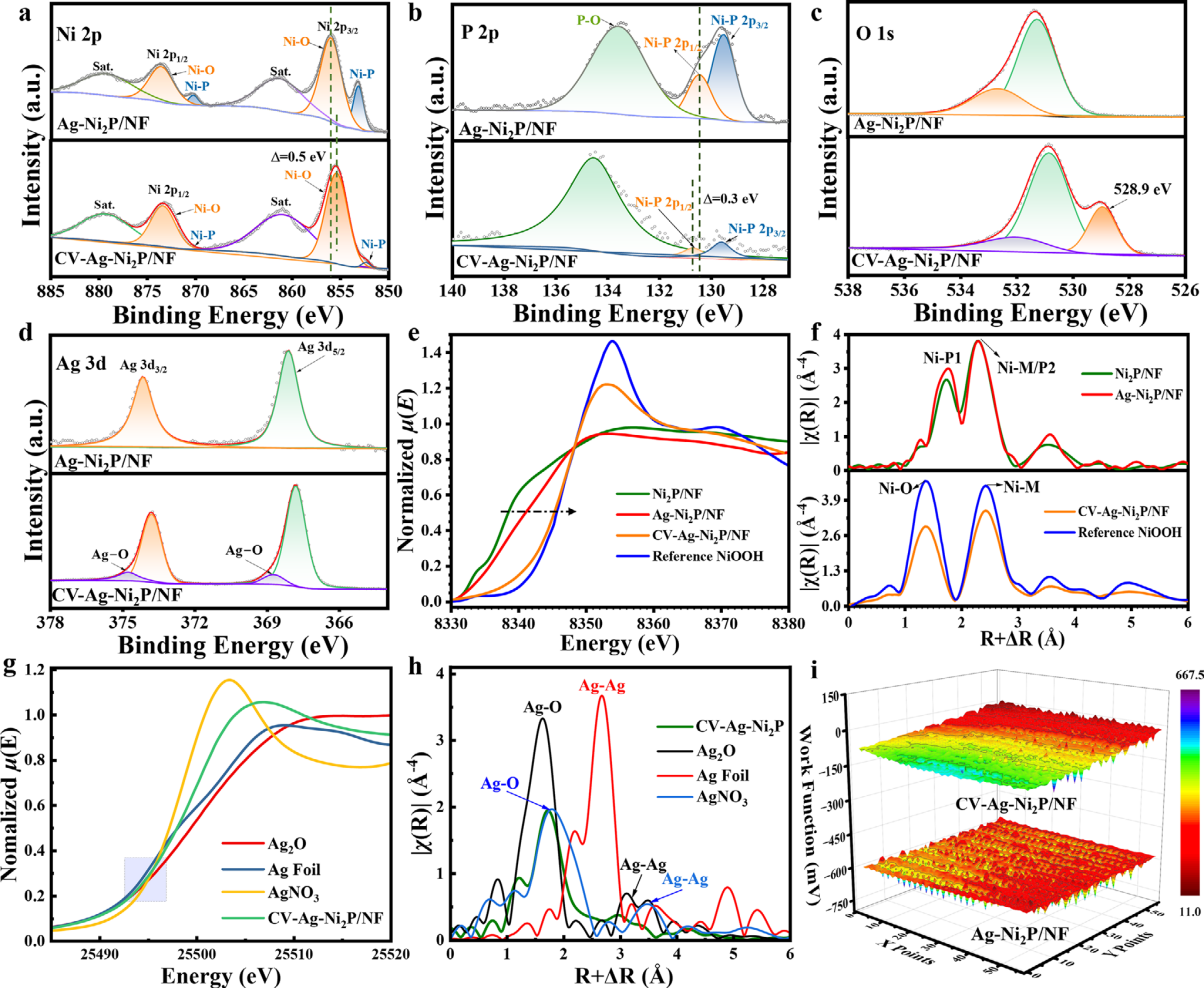
High‐resolution XP spectra of (a) Ni 2p, (b) P 2p, (c) O 1s and (d) Ag 3d in Ag‐Ni_2_P/NF (top panel of the Figures) and CV‐Ag‐Ni_2_P/NF (bottom panel of the Figures), (e) normalized Ni *K*‐edge XANES spectra *µ*(*E*) and (f) FT|*k* ^3^ *χ*(*k*)| spectra without phase correction of Ni_2_P/NF, Ag‐Ni_2_P/NF, CV‐Ag‐Ni_2_P/NF and reference NiOOH, (g) Ag *K*‐edge XANES spectra and (h) EXAFS spectra FT|k^3^χ(k)| of CV‐Ag‐Ni_2_P, metal Ag foil, Ag_2_O and AgNO_3_, (i) KFPM 3D images of Ag‐Ni_2_P/NF and CV‐Ag‐Ni_2_P/NF.

The oxidation states of the samples with or without CV treatment were determined using XPS (Figure 3). The Ni 2p spectra of samples shows two spin‐orbit peaks (Ni 2p_3/2_ and Ni 2p_1/2_). The binding energy of the Ni−O peak in CV‐Ag‐Ni_2_P/NF exhibits a negative shift compared to Ag‐Ni_2_P/NF. This indicates that the electronic interaction with Ni centers has been modulated by CV treatment [27]. In the P 2p spectrum, the P−O peak is dominant in both samples. Meanwhile, Ni−P shows a double‐peak of 2p_1/2_ and 2p_3/2_ at 130.7 and 129.6 eV. After CV treatment, the ratio of Ni−P peaks decreases, further confirming the aforementioned phase evolution. Importantly, in comparison to elemental Ni, the Ni 2p_3/2_ peak of CV‐Ag‐Ni_2_P/NF displays a negative shift (from 855.8 to 855.3 eV), while the P 2p peak moves to a higher binding energy. This suggests partial electron transfer from P to Ni, thus enhancing the electron density around Ni_2_P due to CV treatment (Figure 3a,b) [28, 29]. The O 1s spectrum of CV‐Ag‐Ni_2_P/NF shows a new peak at 528.9 eV and a strong peak at 531.2 eV due to the presence of metal−O and metal−OH after CV treatment, revealing the formation of Ag−Ni oxyhydroxide during the CV process (Figure 3c) [30]. For Ag 3d (Figure 3d), although the peak intensity decreased due to its dissolution in the electrolyte, the remaining Ag signal can still be identified on the catalyst surface. In addition, new peaks at 374.7 and 368.6 eV, corresponding to Ag−O bonds formed after the CV treatment confirmed the transformation of Ag−O_2_ species to (α, β) Ag−O [25]. Similar results can also be extracted from the Raman spectra (Figure 2b). The Ag−Ni oxyhydroxide, metal−O and metal−OH formed by in situ structural reconstruction of Ag‐doped Ni_2_P surfaces are likely to serve as the true catalytic active sites for HER and AOR. In this case, a significantly number of active sites interacting with reactant species would enhance the electrocatalysis process [31].

Those findings were further confirmed by X‐ray absorption near‐edge structure (XANES) spectra *µ*(*E*) (Figure 3e), which show that the energy position of the main absorption edge of the normalized *µ*(*E*) spectrum of CV‐Ag‐Ni_2_P/NF approaches that of reference NiOOH while developing analogous structure features at ∼8353 and ∼8369 eV. Similarly, the Fourier‐Transform FT|*k* ^3^ *χ*(*k*)| spectrum of the extended X‐ray absorption fine structure (EXAFS) spectra *k* ^3^ *χ*(*k*) of CV‐Ag‐Ni_2_P/NF reproduces the main structure peaks of reference NiOOH (Figure 3f), which shows two main peaks at R+ΔR∼1.37 Å and R+ΔR∼2.43 Å arising from single scattering from first and second shell coordinated O and M = Ni/Ag atoms, respectively. These results further indicate that CV‐Ag‐Ni_2_P/NF underwent a structural transformation into NiOOH. The Ag *K*‐edge XANES spectrum of CV‐Ag‐Ni_2_P/NF reveals a near‐edge absorption energy that falls between those of the metal Ag foil and Ag_2_O/AgNO_3_ (Figure 3g), suggesting a partially oxidized Ag valence state, which was determined to be +0.56 from the linear dependence of the Ag *K*‐edge position of the normalized *µ*(*E*) spectra (Figure S6e). Consequently, Ag in Ag‐Ni_2_P exhibits a different electronic environment from that of bulk metallic and fully oxidized Ag species. This was further confirmed by analysis of the EXAFS spectrum, FT|*k^3^χ(k)*|, of Ag‐Ni_2_P, which is different from that of metallic Ag and Ag_2_O/AgNO_3_ (Figure 3h).

Kelvin probe force microscopy (KPFM) (Figure 3i and Figure S7) reveals a pronounced evolution of the surface potential from Ni_2_P/NF to Ag‐Ni_2_P/NF and further to CV‐Ag‐Ni_2_P/NF, indicating that both Ag incorporation and CV activation effectively modulate the local electronic structure and work function. The decreased work function and redistributed surface potential after Ag doping suggest facilitated electron transfer and optimized interfacial charge polarization, while the further electronic reconstruction induced by CV activation is expected to expose more electrochemically accessible active sites and accelerate the adsorption/desorption kinetics of reaction intermediates. These results collectively explain the superior bifunctional activity of CV‐Ag‐Ni_2_P/NF toward both HER and AOR.

### 2.2 Electrocatalytic HER Performance

**FIGURE 4:**
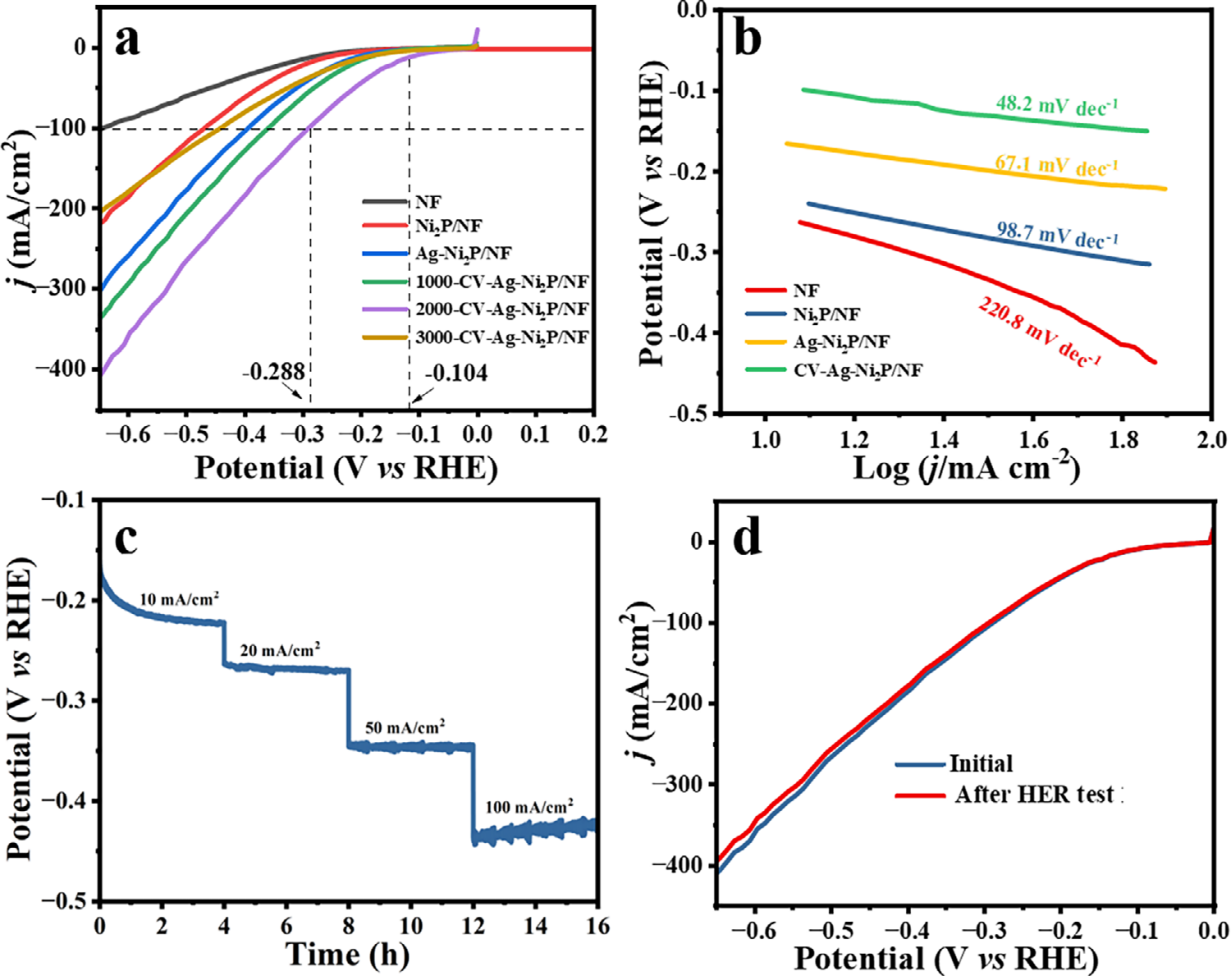
(a,b) LSV curves and Tafel plots of the electrodes in 1 M KOH for HER without IR correction; (c) multi‐current process obtained with the CV‐Ag‐Ni_2_P/NF electrode in 1 M KOH with 10 to 100 mA cm^−2^ of current density; (d) LSV curves for HER for CV‐Ag‐Ni_2_P/NF before and after HER test without IR correction.

The electrocatalytic activity of the samples was investigated using a three‐electrode configuration. Figure 4a displays the relevant LSV curves of the electrodes in 1 M KOH electrolyte for HER activity. It was found that the 2000‐CV‐Ag‐Ni_2_P/NF electrode exhibits higher catalytic activity compared with other electrodes. A potential of 288 mV is required to drive a current density of 100 mA cm^−2^, which is much lower than the corresponding values for Ni_2_P/NF (466.7 mV), Ag‐Ni_2_P/NF (358.5 mV) and 1000‐CV‐Ag‐Ni_2_P/NF (289.5 mV). These results demonstrate that CV treatment significantly promotes HER activity. The intrinsic catalytic activity was evaluated using the corresponding Tafel plots (Figure 4b). The lowest value of the Tafel slope was observed for CV‐Ag‐Ni_2_P/NF (48.2 mV/dec), when compared to that of Ag‐Ni_2_P/NF (67.1 mV/dec) and Ni_2_P/NF (98.7 mV/dec), indicating that both Ag incorporation and CV treatment can enhance the electrocatalytic kinetics. A comparison of the HER overpotential of CV‐Ag‐Ni_2_P/NF with recently reported Ni‐based phosphides and other representative catalysts is given in Table S2. Notably, CV‐Ag‐Ni_2_P/NF exhibits a lower overpotential than most reported phosphide electrocatalysts in 1 M KOH, indicating its competitive HER activity in alkaline media. Furthermore, long‐term chronoamperometry experiments were conducted to test the stability of the CV‐Ag‐Ni_2_P/NF catalyst in 1 M KOH (Figure 4c). The results show that there is no obvious reduction in the electrochemical performance during 16 h of reaction with the current density ranging from 10 to 100 mA cm^−2^, further demonstrating its durability under various current density conditions (Figure 4d).

### 2.3 Electrocatalytic AOR Performance

**FIGURE 5:**
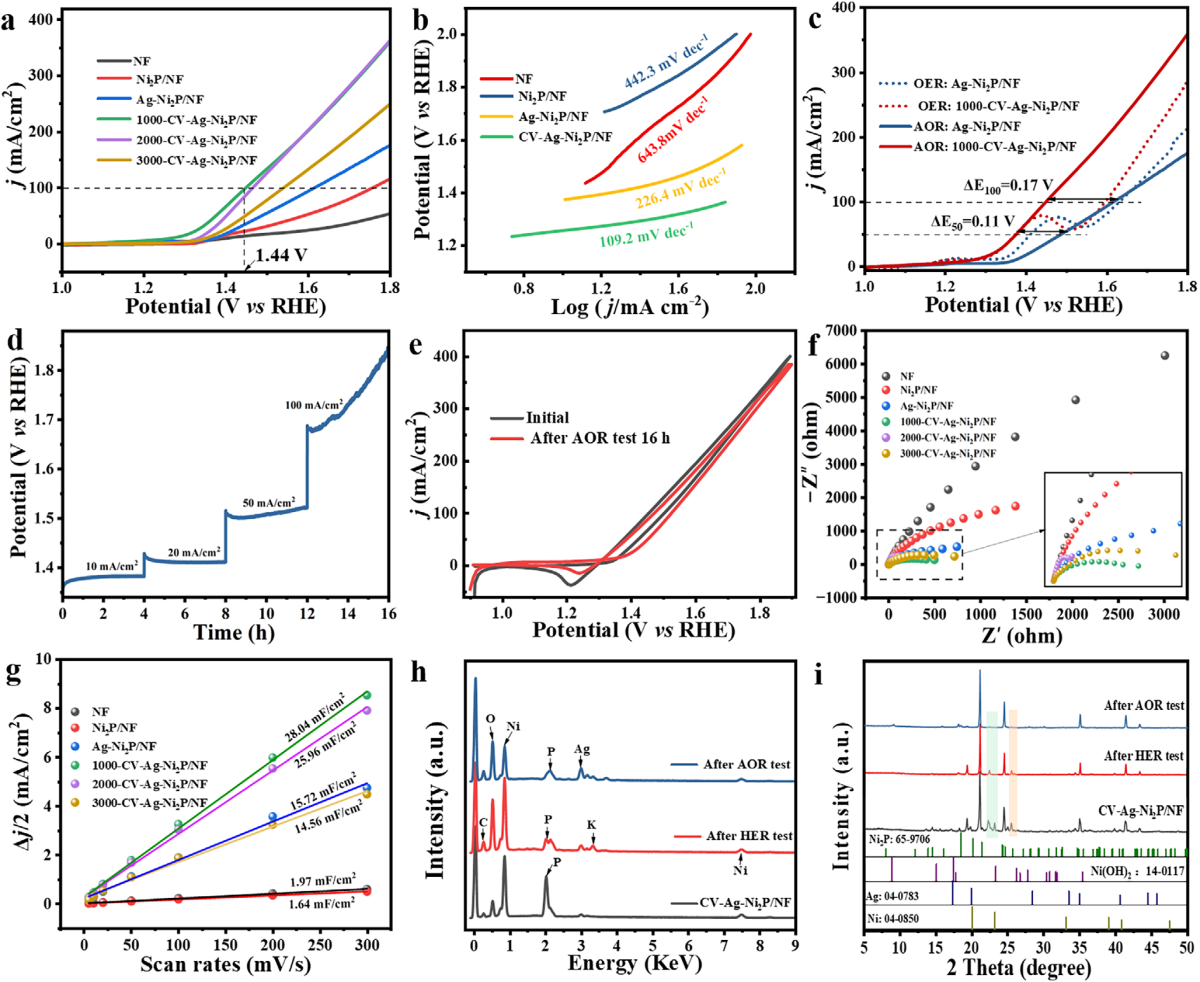
(a,b) LSV curves and Tafel plots of the electrodes in 1 M KOH and 0.5 M AA for AOR without IR correction; (c) AOR and OER of CV‐Ag‐Ni_2_P/NF and Ag‐N_i2_P/NF without IR correction; (d) multi‐current process obtained with the CV‐Ag‐Ni_2_P/NF electrode in 1M KOH and 0.5 M AA with 10–100 mA cm^−2^ of current density; (e) AOR LSV curves of CV‐Ag‐Ni_2_P/NF before and after 16 h test without IR correction; (f) Nyquist plots of catalysts in 1 M KOH; (g) capacitive currents at 0.9 V vs. RHE as a function of scan rates of samples; (h) EDX spectra (i) XRD patterns of CV‐Ag‐Ni_2_P/NF before and after HER and AOR tests.

The AOR catalytic performance of the electrodes was evaluated in 1 M KOH and 0.5 M AA solution. As shown in Figure 5a, at high current density, among the tested samples, 1000‐CV‐Ag‐Ni_2_P/NF exhibited the highest catalytic activity for AOR, demonstrating that CV treatment improves the AOR performance. Only 1.45 V was needed for 1000‐CV‐Ag‐Ni_2_P/NF to achieve the current density of 100 mA cm^−2^. This indicates that structural reconstruction led to increased generation of active species, resulting in an enhancement of the AOR performance. Furthermore, the onset potential value was much lower than those of Ag‐Ni_2_P/NF (1.61 V) and Ni_2_P/NF (1.75 V). As shown in Figure 5b, among all the catalysts, the CV‐Ag‐Ni_2_P/NF electrode had the smallest Tafel slope (109.2 mV/dec), suggesting faster kinetics for AOR with CV‐Ag‐Ni_2_P/NF. To the best of our knowledge, no studies have been reported on AOR tests with CV‐Ag‐Ni_2_P/NF electrodes. The low cell potentials exhibited by CV‐Ag‐Ni_2_P/NF for organic‐assisted water electrolysis compare favorably with other noble‐metal‐free catalysts reported for electrochemical oxidation of organic compounds (Table S3). Our results demonstrate that compared to some reported noble‐metal‐based catalysts, the CV‐Ag‐Ni_2_P/NF catalyst exhibited higher catalytic activity toward the oxidation of small‐molecule organic compounds. From Figure 5c, the potential difference (*Δ*E) between CV‐Ag‐Ni_2_P/NF and Ag‐Ni_2_P/NF is 0.17 V at a current density of 100 mA cm^−2^. It is evident that CV‐Ag‐Ni_2_P/NF exhibits a sharp increase in anodic current, particularly at high voltage when AA is added, indicating that AOR occurs preferentially to OER. However, the current density for CV‐Ni_2_P/NF only slightly differed from Ni_2_P/NF (0.03 V at a current density of 100 mA cm^−2^, Figure S8). This points out the significance of Ag for good conductivity and fast mass transport of the electrode. Moreover, due to the exposure of the active site and its large surface area, Ag plays a crucial role in synergistically reconstructing the active phase and adjusting the electronic structure of CV‐Ag‐Ni_2_P/NF. LSV tests were performed to assess the stability of the CV‐Ag‐Ni_2_P/NF catalyst over 16 h (Figure 5d). The potential for the CV‐Ag‐Ni_2_P/NF remained stable for current densities ranging from 10 to 50 mA cm^−2^. Moreover, as seen in Figure 5d, the potential increased at a current density of 100 mA cm^−2^ due to the rapid and massive degradation of AA and interfacial mass‐transfer limitations at this high current density. The rapid depletion of AA near the electrode surface, together with the accumulation of reaction products/intermediates and enhanced bubble coverage, increases the overall polarization, thereby requiring a higher potential to maintain the constant current. The current density slightly decreased after continuous long‐term operation, further showing the superior stability of the reconstructed catalyst under high current density (Figure 5e). The stability of CV‐Ag‐Ni_2_P/NF for AOR was also tested by 20 000 repeated CV scanning cycles with minimal deviation observed, suggesting the long‐term stability of CV‐Ag‐Ni_2_P/NF (shown in Figure S9).

Further investigations into the catalytic activity of the samples were carried out using electrochemical impedance spectroscopy (EIS). The resulting Nyquist plot is shown in Figure 5f. The smallest arc radius of 1000‐CV‐Ag‐Ni_2_P/NF demonstrates its effectiveness in isolating created electron‐hole pairs and efficiently transmitting charge to the electron acceptors at the interface. The significant enhancement in conductivity could potentially be attributed to the heterointerface effect of Ag and Ni_2_P /NF after CV treatment. The double‐layer capacitance (C_dl_) is illustrated in Figure 5g and Figures S10 and S11. The electrochemically active surface area (ECSA), which is proportional to C_dl_, can serve as a useful measure for evaluating the catalytic activity of the electrodes. Among the samples studied, 1000‐CV‐Ag‐Ni_2_P/NF exhibited the highest C_dl_ (28.04 mF cm^−2^), nearly twice than that of Ag‐Ni_2_P /NF (15.72 mF cm^−2^). This suggests that the CV treatment exposed more effective sites and enlarged the electrochemicall active area, thereby increasing the number of active sites in 1000‐CV‐Ag‐Ni_2_P/NF and enhancing its catalytic activity. These results indicate a significant improvement in electron transport processes. The main reason might be that the active species, which were reconstructed in situ, conferred high electronic conductivity inherited from Ag‐Ni_2_P [32].

To further investigate the stability of the CV‐Ag‐Ni_2_P/NF after long‐term reactions, SEM, EDX elemental mapping images, and XRD were employed (Figure 5h,i and Figures S12–S14) to monitor the samples. As for HER electrolysis, the SEM images show that the initial structure of CV‐Ag‐Ni_2_P/NF was maintained, demonstrating that the spherical morphology did not change after the HER test (Figure S12). In addition, the EDX mapping and XRD structure and morphology are also well maintained, further confirming the long‐term stability of CV‐Ag‐Ni_2_P/NF (Figure 5h,i, Figure S13 and Table S4). Taken all this together, these results suggest that surface oxidation does not significantly affect the structure of the catalyst during HER. During AOR, the reconstruction of Ni_2_P into oxyhydroxide‐rich species alters the local coordination environment of Ag, inducing its redistribution into small Ag‐rich clusters (Figure S13) while preserving its role in modulating the local electronic structure and interfacial kinetics of the reconstructed active phase. The spherical structure of CV‐Ag‐Ni_2_P/NF was retained after the AOR test, however, the outer rod structures within spherical morphology get thicker and develop cracked features due to the etching of the surface by a strong alkaline solution under a high current density (Figure S14). It is clear that the surface of the nanosheets has a significant roughness and is covered with a thin layer of particles, which are caused by the oxidation of the catalyst surface. XRD and EDX analysis were employed to identify the composition of the catalyst. The XRD pattern of CV‐Ag‐Ni_2_P/NF after AOR test shows the attenuation and disappearance of the diffraction peaks of the Ni_2_P‐related crystalline phase (Figure 5i, highlighted green and orange areas). The EDX results (Table S4) indicated a significant increase in the O content of the material, from 12.1% to 33.8%, which is attributed to the oxidation of P to phosphate and oxidation of the catalyst surface to nickel oxide species during the AOR process [33, 34]. As a result, the particles may be composed of oxide species that were generated during the AOR process. Recent studies have indicated that during high potentials under anodic oxidation, electrocatalysts often transform into related (oxy)hydroxides [35]. These species are believed to be the true active sites responsible for the oxidation of small molecular organic matter. Based on the previous results, the formation of Ni (oxy)hydroxide species can be attributed to the in situ electrochemical oxidation occurring on the catalyst surface. Active sites were generated during the AOR process, resulting in high levels of electrochemical activity and stability toward the oxidation reaction. Therefore, the electrocatalytic performance of CV‐Ag‐Ni_2_P/NF stems from a combination of core phosphides and surface‐oxidized species. These findings altogether highlight the remarkable activity of the CV‐Ag‐Ni_2_P/NF catalyst after restructuring for both HER and AOR.

**FIGURE 6:**
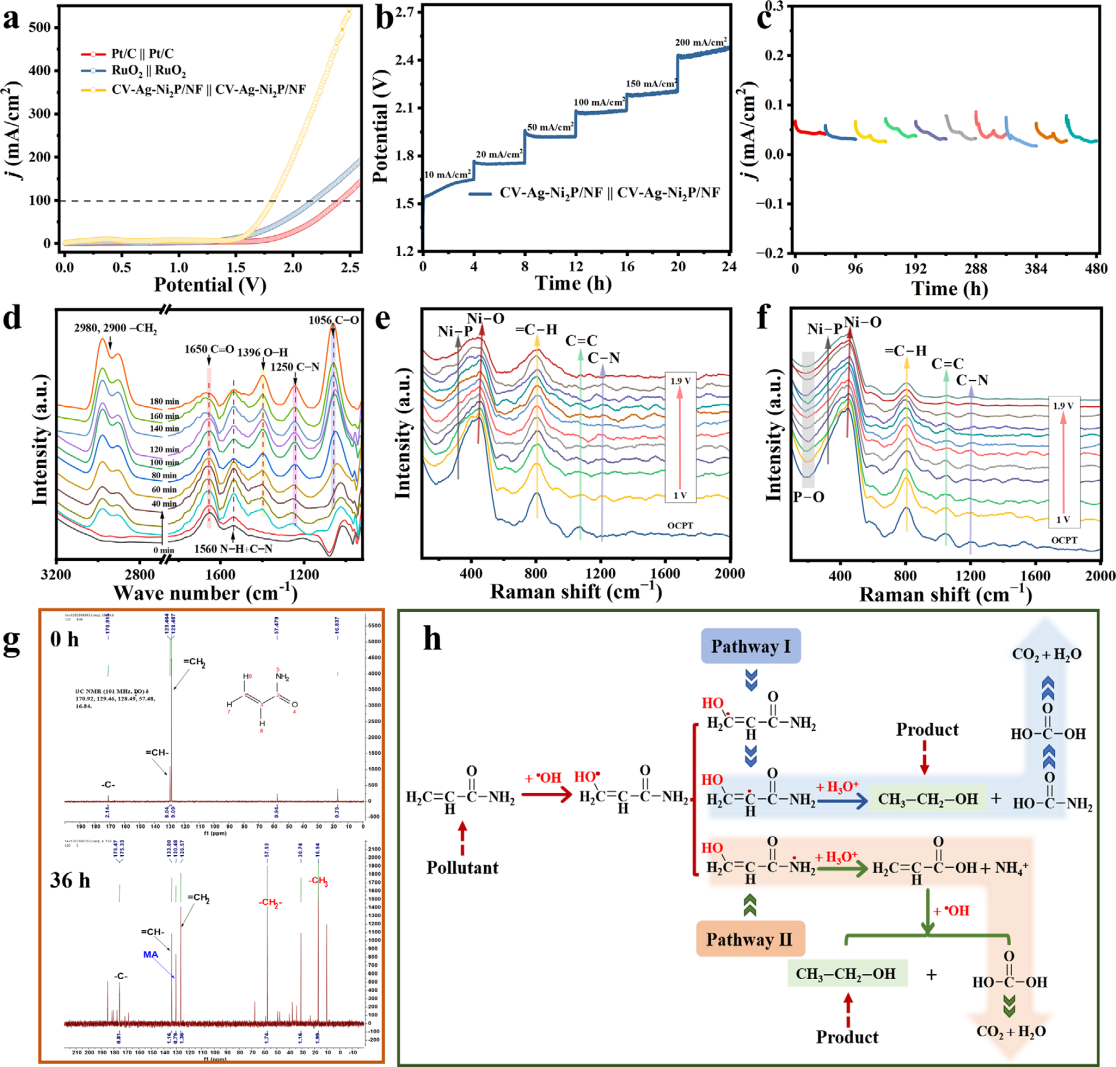
(a) LSV curves of CV‐Ag‐Ni_2_P/NF, RuO_2_/NF and Pt/C/NF electrodes in 1 M KOH and 0.5 M AA for AOR in a two‐electrode cell setup without IR correction; (b) multi‐current process obtained with the CV‐Ag‐Ni_2_P/NF || CV‐Ag‐Ni_2_P/NF electrode in 1 M KOH and 0.5 M AA with 10–200 mA cm^−2^ of current density; (c) chronoamperometry i‐t curve for AOR of CV‐Ag‐Ni_2_P/NF || CV‐Ag‐Ni_2_P/NF electrode; (d) in situ FT‐IR spectra of AA before and after electrolysis; (e,f) in situ Raman spectra of Ni_2_P/NF and CV‐Ag‐Ni_2_P/NF electrode; (g) ^13^C NMR spectrum of AA solution before and after AOR tests; (h) proposed degradation mechanism of AOR.

Due to its high electrocatalytic performance in both HER and AOR, the bifunctional catalyst CV‐Ag‐Ni_2_P/NF was employed as both the cathode and anode in a two‐electrode cell to evaluate its overall catalytic activity (Figure 6a). When compared with commercial RuO_2_/NF and Pt/C/NF catalysts, which require 2.2 and 2.4 V, respectively, to obtain a current density of 100 mA cm^−2^, the CV‐Ag‐Ni_2_P/NF based AA electrolyte only requires 1.52 and 1.8 V to drive current densities of 10 and 100 mA cm^−2^, i.e. lower voltages than those required for RuO_2_/NF and Pt/C/NF. More importantly, at varied current densities from 10 to 200 mA cm^−2^, CV‐Ag‐Ni_2_P/NF‐based AA electrolyte performs with long‐term stability, showing a smooth and stable response even after 24 h (Figure 6b). It is worth noting that the advanced performance of catalysts is a prerequisite for alkaline electrolyzers with a current density of 10–200 mA cm^−2^, demonstrating CV‐Ag‐Ni_2_P/NF as a potential candidate for industrial production [36]. Furthermore, the long‐term durability of CV‐Ag‐Ni_2_P/NF||CV‐Ag‐Ni_2_P/NF was further assessed by a 20‐day electrolysis test under AOR conditions (Figure 6c). To eliminate the influence of AA consumption during prolonged operation, the electrolyte was renewed every 2 days with fresh AA‐containing solution. The catalyst exhibits a stable current response over 480 h, evidencing excellent operational stability. It is safe to say that the durability of the catalyst and the fast mass transport at high current density promote the oxidation rate of AA. After AOR tests at 50 mA cm^−2^ for 24 h, AA degradation reached approximately 80% and the changes in the solution UV spectra over the reaction time are shown in Figures S15 and S16.

### 2.4 Mechanisms for Acrylamide Degradation

In situ FT‐IR spectra of the solution before and after the AOR were recorded to gain a deeper understanding of AA degradation process (Figure 6d and Figure S17). The peaks at 1650 and 1560 cm^−1^ are attributed to C═O and C─N bonds, respectively [37]. The intensities of these characteristic peaks were significantly reduced, and even disappeared after AOR, indicating that the amide group in the AA molecule was oxidized. The signal at 1119 cm^−1^, corresponding to the −CH_3_ bending vibrations of −N^+^(CH_3_)_3_, also disappeared after AOR, indicating that the main chain was broken and trimethylamine group (−N^+^(CH_3_)_3_) was degraded [38]. The in situ FT‐IR spectra shows intensified absorption bands corresponding to −CH_2_− stretching vibrations (2800–2900 cm^−1^), O−H bending (1396 cm^−1^), and C−O stretching (1056 cm^−1^) following prolonged AOR test (Figure S17). However, the band at 3300 cm^−1^, which is assigned to the strong stretching oscillation of –N–H and O–H in COOH and –CONH_2_, still remains significant for the solution [37], revealing that only some acrylamino groups resulted from ammonia hydrolysis. Therefore, the FT‐IR results suggested that the CV‐Ag‐Ni_2_P/NF effectively degrades AA to produce ethanol.

Operando Raman spectroscopy reveals a clear potential‐driven surface reconstruction of both Ni_2_P/NF and CV‐Ag‐Ni_2_P/NF in alkaline electrolyte. With increasing anodic bias, gradual spectral evolution is observed in the low‐wavenumber (∼440–460 cm^−^^1^) and ∼800 cm^−^^1^ regions, indicating the formation of a reconstructed Ni oxyhydroxide‐rich surface derived from the phosphide precursor (Figure 6e,f). Notably, no discernible variation in the acrylamide‐related Raman bands is observed within −0.1 to −0.6 V vs. RHE, indicating that AA remains essentially unreactive at low bias (Figure S18). Upon further increasing the applied potential (1.0–1.9 V vs. RHE), CV‐Ag‐Ni_2_P/NF exhibits a much more pronounced attenuation and evolution of the AA‐associated bands than pristine Ni_2_P/NF, mainly arising from the amide C–N and C═C containing framework, suggesting that Ag incorporation markedly facilitates AA adsorption/activation and subsequent bond cleavage on the reconstructed surface (Figure 6e,f). Collectively, these findings highlight the synergistic effect of Ag doping and CV‐induced surface reconstruction in promoting AA oxidation kinetics and enabling efficient electrochemical degradation.

To explore the degradation mechanism of AA, ^13^C NMR and ^1^H NMR were performed to verify the identified composites in solution before and after degradation. According to Figures S19 and S20 and Figure 6g, AA carbon signals were identified by pairing with the standard signals. The spectra exhibit erroneous signals likely originating from reagent impurities, including residual starting materials or synthetic byproducts. However, the carbon signals (C, = CH_2_, = CH−) corresponding to AA were determined to be robust and reliable throughout initial characterization. After 2 h of AOR treatment, the = CH_2_ and = CH− signal intensities showed significant attenuation, concurrently with the emergence of intermediate product signals. Notably, after 24 h of reaction, carbon signals assigned to ethanol (−CH_2_−, −CH_3_) were identified in the AOR‐treated solution, while those corresponding to AA (C, = CH−) were significantly diminished. In addition, the two carbon signals found in the solution after AOR tests were validated by comparing them with the ^13^C NMR of standard ethanol signals (Figure S19b). To confirm the presence of ethanol, we compared the observed ^1^H NMR signals with the ^13^C NMR in the AOR‐treated solution. The hydrogen signals (H_6_, H_7_, H_8_,) corresponding to AA, the presence of hydrogen signals (−CH_2_−, −CH_3_) in the solution after the AOR test, which matched those of ethanol, further supported the degradation of AA and the generation of ethanol. The concentration of ethanol was quantified via ^1^H NMR spectrum by comparing the integral of its methyl group (−CH_3_) resonance to that of the maleic acid (MA) internal standard. Ethanol concentration increased with reaction time: 0.0017 M (2 h), 0.0084 M (6 h), 0.0278 M (24 h), 0.0753 M (36 h). To clarify the carbon fate during AOR, TOC analysis was performed together with NMR quantification (Figure S21). After 24 h electrolysis, the TOC decreased from 15 355 to 10 850 mg L^−^^1^, corresponding to a net loss of 4970 mg L^−^^1^ dissolved organic carbon, while ^1^H NMR confirmed ethanol formation at 0.0278 M (∼667.2 mg L^−^^1^ carbon). These results suggest that, in addition to ethanol generation, a substantial fraction of AA‐derived carbon was further oxidized, most likely into bicarbonate/carbonate species under alkaline conditions. HR‐ESI‐MS reveals the disappearance of the characteristic AA peak and the emergence of new low‐retention‐time products after AOR, indicating that AA is converted into smaller oxygenated molecules rather than undergoing simple functional‐group transformation (Figure S22).

Based on the above analysis, AA degradation during the AOR is suggested to proceed predominantly through a radical‐induced chain‐scission pathway. To clarify the effect of •OH on the AA degradation, AA was degraded in the presence of free radical scavenging agents (nitrobenzene, NB) [39]. Figure S23 shows the inhibition effects of radical scavengers on AA oxidation by AOR test. When 0.2 mmol/L NB was added, the removal of AA decreased by 49.1%. The results show that •OH played an important role in AA degradation. A detailed reaction scheme for this degradation pathway is presented in Figure 6h [40, 41, 42, 43, 44]. The degradation of AA initially involves the hydroxyl radical (•OH) attacking the α‐position C, which generates AA radicals (Equations (1), (2), (3)). Previous studies identified three possible radical sites that can react either with each other or with other radicals [45]. The generation of acrylamide radicals occurs simultaneously and can undergo transformations through these radical sites. These transformations may involve a series of chemical reactions, including but not limited to hydrogen abstraction, addition, and elimination [46]. Pathway I: acrylamide radicals (${\mathrm{HOH}}_{2}C=\dot{\mathrm{CH}}\;{\mathrm{CONH}}_{2}$) react with hydronium ion (H_3_O^+^) to generate CH_3_CH_2_OH and HOCONH_2_ (Equation 4), then HOCONH_2_ continues to react to H_3_O^+^ to form HOCOOH and NH_4_^+^ (Equation 5), finally, HOCOOH decomposes to form H_2_O and CO_2_. At the same time, pathway II: another acrylamide radical (${\mathrm{HOH}}_{2}C=\mathrm{CHCO}\dot{N}H_{2}$) also reacts with H_3_O^+^ to form H_2_C  =  CHCOOH and NH_4_^+^ (Equation 6), and H_2_C  =  CHCOOH can be attacked by •OH to continue the chemical degradation to form $H_{2}C\dot{C}\mathrm{COOH}$ (Equation 7), then $H_{2}C\dot{C}\mathrm{COOH}$ reacts to H_3_O^+^ to form CH_3_CH_2_OH and HOCOOH (Equation 8), Finally, HOCOOH will further decompose to H_2_O and CO_2_ (Equation 9). At last, these small molecules are all oxidized into methanol and NH_4_^+^ in the aqueous solution. Through these reactions, the AA molecule gradually degrades into various intermediate products until it is ultimately converted into simpler, harmless end products. The results obtained suggest that AOR has the potential to replace OER in electrochemical systems. This substitution could not only further reduce the cell voltage of HER, but also lead to the production of high‐value‐added methanol at the anode. Furthermore, the electrochemical process involved in AOR operates under mild conditions, making it environmentally friendly and cost‐effective.

(1) $$H_{2}C=\mathrm{CHCON}H_{2}+•\mathrm{OH}\to \mathrm{HO}H_{2}\dot{C}=\mathrm{CHCON}H_{2}$$

(2) $$H_{2}C\;=\mathrm{CHCON}H_{2\;}+•\mathrm{OH}\;\to \mathrm{HO}H_{2}C=\mathrm{CHCON}H_{2}$$

(3) $$H_{2}C=\mathrm{CHCON}H_{2}+•\mathrm{OH}\;\to \mathrm{HO}H_{2}C\;=\mathrm{CHCO}\dot{N}H_{2}$$

(4) $$I:\mathrm{HO}H_{2}C=\dot{C}\mathrm{HCON}H_{2}+H_{3}O^{+}\to {\mathrm{CH}}_{3}{\mathrm{CH}}_{2}\mathrm{OH}\;+\mathrm{HOCON}H_{2}$$

(5) $$\mathrm{HOCON}H_{2}+H_{3}O^{+}\to \mathrm{HOCOOH}\;+{{\mathrm{NH}}_{4}}^{+}$$

(6) $$\mathrm{II}:\;\mathrm{HO}H_{2}C\;=\mathrm{CHCO}\dot{N}H_{2}+H_{3}O^{+}\;\to H_{2}C=\mathrm{CHCOOH}\;+N{H_{4}}^{+}$$

(7) $$H_{2}C=\mathrm{CHCOOH}\;+•\mathrm{OH}\;\to H_{2}C\dot{C}\mathrm{COOH}\;+H_{2}O$$

(8) $$H_{2}C\dot{C}\mathrm{COOH}+H_{3}O+\to CH_{3}CH_{2}\mathrm{OH}+\mathrm{HOCOOH}$$

(9) $$\mathrm{HOCOOH}\;\to CO_{2}+H_{2}O$$

### 2.5 Density Functional Theory (DFT) Calculations

**FIGURE 7:**
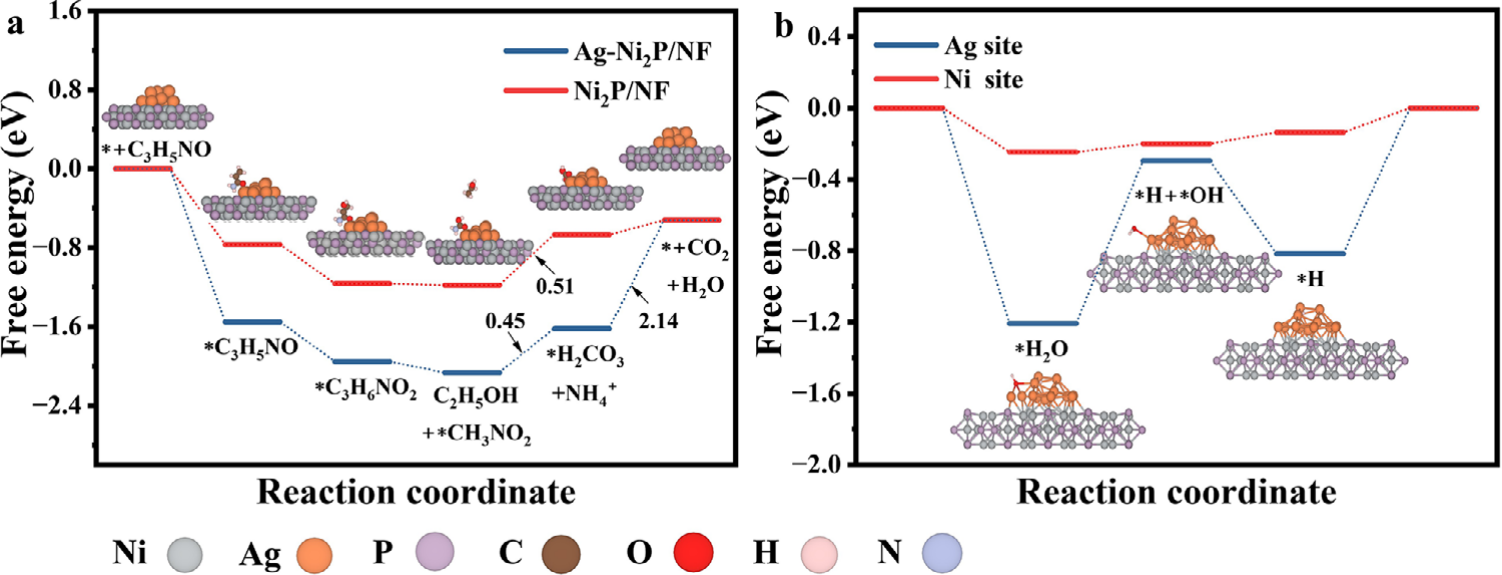
(a) Calculated free energy diagrams for AOR reaction intermediates adsorbed on Ni_2_P/NF and Ag‐Ni_2_P/NF; (b) calculated free energy diagrams of HER for Ag‐Ni_2_P/NF on different positions. Color code: gray, Ni; orange, Ag; purple: P; brown, C; red, O; pink H; blue, N.

Efficient degradation of AA requires a well‐designed catalytic surface. To explore the driving force of CV‐Ag‐Ni_2_P/NF for HER and AOR, we simulated the processes of AA degradation and water splitting on in situ reconstructed catalyst surfaces. The strong metal‐support interaction stabilizes Ag nanoclusters on the Ni_2_P surface, providing efficient adsorption sites for AA. Figure 7a and Figure S24 depict the five‐step degradation pathways of an AA molecule and the relative free energy profiles from AA to H_2_O and CO_2_, which is used to illustrate the mechanism for the better performance of Ag‐Ni_2_P/NF. Compared to the free energy required to adsorb AA on the Ni_2_P surface (ΔE = −0.76 eV), the adsorption of AA on the Ag clusters on the Ag‐Ni_2_P surface is stronger (ΔE = −1.55 eV), suggesting that the first adsorption process is thermodynamically spontaneous. During AA decomposition, charge transfer drives the binding of the OH group to α position C, generating AA radicals. The step with the highest barrier is considered the potential‐determining step for the AOR process. The intermediate product (*C_3_H_6_NO_2_) is a stable molecular structure that drives the degradation of AA with reduced free energy, as indicated by a calculated ΔE of −0.40 eV. Hydronium ions (H_3_O^+^) generated from water splitting react with the AA intermediate to induce C−chain scission, where H^+^ combines with β position C to split off ethanol (CH_3_CH_2_OH), and OH^−^ adsorbs at the C−C bond break to generate HOCONH_2_. The production of ethanol is facilitated on the Ag cluster surface with ΔE = −0.11 eV. The residual free radical NOCONH_2_ is further degraded into H_2_CO_3_ and NH_4_^+^ during water splitting, enabling environmentally friendly degradation of harmful molecules in the electrocatalytic AOR process. In addition, the calculation results suggest that the desorption of *CH_3_NO_2_ to *H_2_CO_3_ and *H_2_CO_3_ to H_2_O + CO_2_ on the surface of Ag‐Ni_2_P/NF requires energies of 0.45 and 2.14 eV, respectively. This also provides a thermodynamic possibility for the oxidation of AA to produce ethanol, rather than being completely oxidized to H_2_O and CO_2_. The findings demonstrate the importance of rationally designed catalytic surfaces in achieving efficient electrocatalytic AOR kinetics. Ag clusters on the Ni_2_P surface serve as active sites for AA degradation and have the potential to be a prefer catalytic sites for electrocatalytic AOR.

Efficient water splitting is crucial for investigating the catalytic activity in the alkaline HER process. The adsorption capacity of water molecules on different metal sites was taken into consideration and shown in Figure 7b and Figure S25. It is widely recognized that the first step in HER in alkaline media involves the adsorption and dissociation of the H_2_O on the catalyst surface and is often regarded as the kinetic limiting step [33, 47]. The free energy level diagram shows that Ag sites have a higher H_2_O adsorption energy compared to the Ni sites, indicating a faster H_2_O‐adsorption on the surface of the Ag‐Ni_2_P, which is favorable to the subsequent splitting step. The water splitting process (H_2_O* → H*+OH*) on Ag sites, which requires 0.91 eV, presents a more significant challenge. However, H_2_O molecules adsorbed on Ni sites undergo easier splitting into hydroxyl groups and protons (0.05 eV), with OH^−^ remaining on the original Ni site, while H^+^ detaches and adsorbs onto another Ni site. The lower free energy (0.14 eV) required for H^+^ desorption from Ni sites, which is significantly lower than that from Ag sites (0.82 eV), indicates that Ni sites can effectively facilitate water dissociation and enhance the kinetics of HER on the surface of the Ag‐Ni_2_P catalyst [48]. The subsequent desorption of H_2_ molecules then occurs from active Ni sites. Although the Ag sites reveal weak binding energy for water splitting and H^+^ desorption, which is unfavorable for catalytic performance, they do exhibit higher H_2_O adsorption energy. This suggests that the synergistic effect of Ag and Ni sites can optimize the adsorption energy of Ni_2_P with the introduction of Ag. The above DFT calculation revealed that the active metal sites of the Ag‐Ni_2_P catalyst fulfill their respective roles in the HER and AOR processes. Ag sites efficiently degrade AA, while Ni sites drive HER with a low overpotential.

## 3 Conclusion

In summary, by combining multiple characterization techniques with in situ spectroscopic analysis, this work elucidates the electrocatalytic oxidation pathway of AA and proposes plausible reaction steps on Ag‐doped Ni_2_P nanosheets on Ni foam followed by an in situ CV treatment (CV‐Ag‐Ni_2_P/NF). Moreover, the results demonstrate the potential of electrocatalytic AOR as a practical strategy for wastewater treatment and promoting HER. The CV‐Ag‐Ni_2_P/NF requires only 1.52 and 1.82 V to reach current densities of 10 and 100 mA cm^−2^, which shows an obviously superior performance than RuO_2_/NF and Pt/C/NF catalysts (2.2 V and 2.4 V, respectively, to obtain the same current densities), with a remarkable Faraday efficiency to produce ethanol (79.3%). In situ FT‐IR and Raman spectra revealed the dehydrogenation and denitrogenation steps in the AOR process and predicted ethanol as the main oxidation product. With the results of DFT calculations, it is revealed that the catalytic performance of CV‐Ag‐Ni_2_P/NF can be attributed to the optimization of the electronic structure of Ni_2_P by Ag doping, which generates an optimistic site or H* absorption and lowered the dehydrogenation energy of AA in AOR. After long‐term HER and AOR tests on CV‐Ag‐Ni_2_P/NF, post‐characterizations (XRD, SEM, and EDX mapping) also confirmed the long‐term performance stability of the reconstructed electrode. Our coupled AOR strategy for promoting HER conveniently decreases energy consumption and affords high‐value‐added ethanol, which provides a clear and promising perspective for AOR.

## 4 Experimental Section

### 4.1 Chemicals

Ammonium fluoride (≥98.0%), Ni(NO_3_)_2_·6H_2_O (≥99.9%), AgNO_3_ (≥99.0%), potassium hydroxide (≥85.0%), sodium hypophosphite (98%), urea (≥99.0%), IrO_2_ (99.9%), RuO_2_ (99.9%), acrylamide (99.0%), and 20 wt.% Pt/C were purchased from Sigma‐Aldrich. Ni foams were purchased from Kunshan Electronic Materials Co., Ltd (Kunshan, China). All chemicals were used as received without any further purification. Deionized water was used for all experiments.

### 4.2 Preparation of Ag_n_Ni_1−n_(OH)_x_/NF Electrodes

First, Ni foam (NF, 15 × 30 × 0.5 mm) was cleaned with 3.0 M HCl and deionized water for 15 min in an ultrasonic bath. Ag_n_Ni_1−n_(OH)_x_/NF was synthesized by a hydrothermal method. In short, the desired amounts of Ni(NO_3_)_2_·6H_2_O, AgNO_3_, urea (11.65 mmol), and NH_4_F (5.41 mmol) were dissolved in deionized water (80 mL). The total amount of metal ions was kept at 3 mmol, and different molar ratios of Ag:Ni were used to prepare Ag_n_Ni_1−n_/NF (*n* = 0, 0.33, 0.5, 0.67, and 1). Afterward, the above solution was transferred into a 15 mL Teflon‐lined autoclave and heated at 180°C for 12 h. The as‐prepared samples were washed by deionized water and dried at 60°C for 10 h. Ar gas used in the synthesis was purchased from PanGas AG, Switzerland, ≥99.999%.

### 4.3 Preparation of Ag‐Ni_2_P/NF Electrodes

The as‐prepared Ag_n_Ni_1−n_(OH)_x_/NF and NaH_2_PO_2_ powder (500 mg) were placed at two separate crucibles with NaH_2_PO_2_ on the upstream side of the tube furnace. The samples were annealed at 320°C for 2 h with a ramping rate of 5°C min^−1^ under Ar atmosphere. The mass loading was determined to be ≈1.2 mg cm^−2^, by measuring the weight difference between pure NF and NF covered with catalysts. Ag_n_Ni_1−n_(OH)_x_/NF electrodes were used in the following sections unless otherwise stated because as they showed the highest OER and AOR catalytic performances, respectively (Figure S2).

### 4.4 Preparation of CV‐Ag−Ni_2_P/NF Electrodes

The obtained electrodes were reconstructed in situ by cyclic voltammetry (CV). The CV‐Ag−Ni_2_P/NF electrodes were cycled in 1 M KOH solution from 0 to 0.8 V vs. Hg/HgO for 1000, 2000, and 3000 cycles at a scan rate for 100 mV/s. The samples with different cycles of CV (1000, 2000, and 3000 cycles at a scan rate for 100 mV/s) were denoted as 1000‐Ag‐Ni_2_P/NF, 2000‐Ag‐Ni_2_P/NF, and 3000‐Ag‐Ni_2_P/NF.

### 4.5 Preparation of RuO_2_/NF and Pt/C/NF With Commercial Catalysts

20.0 mg of commercial RuO_2_ and 20% Pt/C were dispersed in the mixture of deionized water (375 µL), ethanol (125 µL), and 5 wt.% Nafion (50 µL) after sonication for 30 min to form a homogeneous ink. Next, the catalyst ink was drop‐casted onto NF substrates to prepare the samples (loading amounts of 2 mg/cm^2^). The electrodes were dried at room temperature overnight before use.

### 4.6 Materials Characterization

Electrode surface morphologies were characterized by scanning electron microscopy (SEM, Carl ZEISS AG, Merlin compact, Germany) and a high‐resolution transmission electron microscopy (HRTEM, FEI Tecni G30, USA). Powder X‐ray diffraction (PXRD) patterns were recorded on a STOE STADI P diffractometer (transmission mode, Ge monochromator) with Mo K_α_ (λ = 0.7093 Å) radiation operated at a voltage of 50 kV and a current of 40 mA. Fourier‐transform infrared (FT‐IR) spectra were recorded on a Bruker Vertex 70 spectrometer equipped with a Platinum ATR accessory containing a diamond crystal. For the in‐situ FT‐IR testing, we employed 20 mL of electrolyte solution (1.0 M KOH and 0.5 M acrylamide) with a 5 mm × 10 mm × 0.5 mm CV‐Ag‐Ni_2_P/NF working electrode and a Pt counter electrode. In situ Raman spectra were recorded on a LabRAM HR Evolution Raman spectrometer (Horiba) using a 532 nm excitation laser with the laser power set at 5%. An in situ electrochemical cell was employed for the measurements, using a Pt wire as the counter electrode, the CV‐Ag−Ni_2_P/NF electrode as the working electrode, and the 1 M KOH/0.5 M AA mixed solution as the electrolyte. Raman spectra were recorded at different applied potentials to monitor the potential‐dependent evolution of surface species during the electrochemical process. For Kelvin probe measurements, conductive Si wafers were first rinsed with ethanol and dried in air. Catalyst inks were prepared by dispersing 2 mg of powder in 1 mL of isopropanol/water (9:1, v/v), followed by ultrasonication for 20 min. The suspension was drop‐cast onto the Si wafer in 1–2 µL aliquots with multiple depositions to obtain a thin but continuous particle layer. Surface potential measurements were then conducted on a scanning Kelvin probe system (SKP5050, KP Technology) under ambient conditions. High‐Resolution Electrospray Ionization Mass Spectrometry (HR‐ESI‐MS) spectra were acquired on a Bruker tims TOF Pro mass spectrometer in positive ESI mode. The sample was prepared in MeOH and introduced by syringe pump. The mass range was m/z 50–3000. The ESI parameters were set as follows: capillary voltage 4500 V, end‐plate offset −500 V, nebulizer gas pressure 0.4 bar, dry heater 200°C, and dry gas flow 3.5 L min^−^^1^. The measurements were conducted at a current density of 50 mA/cm^2^. X‐ray photoelectron spectroscopy (XPS) spectra were recorded on a PerkinElmer PHI 1600 ESCA system with an excitation radiation source of Mg K_α_ 1253.6 eV. Raman spectra were recorded on a Renishaw Raman scope or InVia Qontor (Ar+ laser, 532 nm). Brunauer‐Emmett‐Teller (BET) surface areas of the samples were measured with a surface area analyzer. The static capacity method was used for BET tests, and the pretreatment conditions were 120°C min^−1^, with nitrogen used as the adsorption gas, and the adsorption temperature set at 77 K (JW‐BK 122 W, Beijing JWGB Sci. & Tech. Co., Ltd., China). ^13^C and ^1^H nuclear magnetic resonance (NMR) spectra were recorded on a BRUKER AVANCE 3–400 spectrometer. For quantitative NMR analysis, in a 5 mm NMR tube, 200 µL of the sample, 100 µL of a 0.05 M Maleic acid (MA) solution (internal standard), and 200 µL of deuterium oxide (D_2_O) were added as a signal locking solvent. For the analysis of acrylamide (0.5 M) and ethanol (purity) solutions, in a 5 mm NMR tube, 200 µL of the solution was combined with 300 µL of D_2_O were added as a signal locking solvent. The concentration of ethanol was determined by comparing the integral of its methyl group (‐CH_3_) resonance to that of the MA internal standard in the ^1^H NMR spectrum.

### 4.7 Electrochemical Measurements

Electrochemical tests were performed in a standard three‐electrode test system in 1 M KOH electrolyte on a Bio‐Logic SP‐150 electrochemical workstation. As‐prepared samples served as the working electrode, with Pt plate and Hg/HgO electrode as the counter and reference electrodes, respectively. The exposed surface area of the working electrode was 1 cm^2^. All of the catalyst electrodes were continuously scanned for ten times with CV before measuring the polarization curves. Linear sweep voltammetry (LSV) and CV were performed at a scan rate of 2 mV/s after ten activating CV cycles without IR compensation. Tafel slopes were acquired from the LSV curves according to the equation:

$$\eta =b\;\log\;\left|j\right|+a$$

where η denotes overpotential, j denotes current density, b denotes Tafel slope and a denotes intercept.

Electrochemical impedance spectroscopy (EIS) measurements were performed at open circuit voltage from 0.01 Hz to 100 kHz with 5 mV of amplitude. The double‐layer capacitances (C_dl_) of samples were obtained by CV measurements to assess the electrochemically active surface area (ECSA). The long‐time stability and durability were evaluated by chronopotentiometry (i‐t) and CV methods. All potentials measured in alkaline media were calibrated to the reversible hydrogen electrode (RHE) using the following equation:

$$E_{\mathrm{RHE}}=E_{\mathrm{Hg}/\mathrm{HgO}}+0.098+0.059\;\times \mathrm{pH}\;\left(\mathrm{where}\;\mathrm{pH}\;=13.6\right)$$

With regard to overall acrylamide‐water splitting, the assembled CV‐Ag‐Ni_2_P/NFǁCV‐Ag‐Ni_2_P/NF couple was used in a two‐electrode configuration with 1.0 M KOH and 0.5 M acrylamide.

### 4.8 X‐Ray Absorption Spectroscopy (XAS)

X‐ray absorption near‐edge structure (XANES) and extended X‐ray absorption fine structure (EXAFS) spectra at the Ni *K*‐edge of Ni_2_P/NF, Ag‐Ni_2_P/NF, and CV‐Ag‐Ni_2_P/NF powder samples dispersed in cellulose were collected using a bench‐top easyXAFS300 spectrometer with a spherical Johann‐style optical analyzer. Measurements were performed in transmission mode using a Mo anode 1200 W X‐ray tube (25 kV; 15 mA), a SDD detector, a He‐filled flight path to reduce air absorption, and a Si(111) spherically crystal analyzer (*hkl*; 444). 10 scans were collected for each sample and merged into a single spectrum, while the scan of a blank cellulose pellet was subtracted for background correction. Energy calibrations were performed using a reference Ni foil. The spectra were reduced into the range Δ*k* ≈3–10 Å^−1^ and Fourier‐Transformed to FT|*k* ^3^χ(*k*)| into the real‐space interval ΔR ≈0–6 Å using ATHENA [1]. All the FT|*k* ^3^χ(*k*)| spectra are presented without phase correction.

### 4.9 Determination of Reactive Species

To test the role of different free radicals during AA degradation, degradation kinetic experiments were tested in the presence of free radical quenchers. Nitrobenzene (NB, 0.25 mmol/L) was added into the reaction solutions at the same time as a trapping agent to scavenge the residual •OH with a rate constant of 3.2 × 10^9^ M^−1^ s^−1^ [2]. In order to determine the mechanism of AA degradation reaction and the contribution of •OH radicals, a certain amount of NB (0.25 M) and AA (0.5 M) was added to the 1 M KOH solution to create a mixed system.

### 4.10 Density Functional Theory Calculations

The structures of Ag‐Ni_2_P and Ni_2_P were constructed to investigate the adsorption behavior of electrochemical AOR and HER in alkaline media. The Projector‐augmented wave (PAW) methodology was utilized to simulate the relationship between valence electrons and cores in the database. To explore the transfer and association of electrons, we adopted Perdew‐Burke‐Ernzerh (PBE)  for exchange‐correlational function and Generalized‐Gradient Approximation (GGA). To expand the plane wave, the energy cutoff value of 450 eV was selected, and a Monkhorst‐Pack grid with a 1 × 1 × 1 sampling was set for the first Brillouin zone. The occupancy was determined by setting a Gaussian smearing width of 0.1 eV. In addition, a vacuum layer was applied to avoid the interaction in the z‐direction of the slab and was positioned in the middle of the top to the bottom of the model, at least 30 Å. Structural relaxation of the adsorption surface was calculated using a conjugate gradient algorithm, and the maximum force and energy of unconstrained atoms were constrained to be less than 0.01 eV/Å and 1 × 10^−5^ eV, respectively.

The adsorption energy (E_ads_).of AOR and HER process is obtained by:

$$\Delta E_{\mathrm{ads}}=E\left(\mathrm{ad}+\mathrm{sub}\right)-E_{\mathrm{ad}}-\;E_{\mathrm{sub}}$$

The adsorption‐free energy (ΔG_ads_) of AOR and HER process is obtained by:

$$\Delta G_{\mathrm{ads}}=\Delta \mathrm{eads}\;+\Delta \mathrm{ZPE}-T\Delta S$$

where ΔZPE and ΔS are the contributions to the free energy from the zero‐point vibration energy and entropy, respectively.

## Conflicts of Interest

The authors declare no conflicts of interest.

## Supporting information




**Supporting File**: smll74237‐sup‐0001‐SuppMat.docx.

## Data Availability

The data that support the findings of this study are available from the corresponding author upon reasonable request.
