# Supplementary material for: In Situ Reconstruction of Ag‐Doped Ni2P Electrocatalyst for Hydrogen Evolution and Value‐Added Acrylamide Conversion
Source: Small. 2026 Jul 14;22(45):e74237. doi: 10.1002/smll.74237 (PMC13472254; doi:10.1002/smll.74237)
Supplement: Supplementary file 1 [file SMLL-22-e74237-s001.docx]

Supporting Information

**In situ reconstruction of Ag-doped Ni_2_P electrocatalyst for hydrogen evolution and value-added acrylamide conversion**

Dan Zhang ^a,b,c^, Zeyi Zhang ^a^, Lingshen Meng ^a^, Carlos A. Triana ^a^, Shangkun Li ^a^, Hang Chen ^a^, Yiming Li ^b^* and Greta R. Patzke ^a*^

^a^ Department of Chemistry, University of Zurich, Winterthurerstrasse 190, CH-8057 Zurich, Switzerland.

^b^ China Frontiers Science Center for Deep Ocean Multispheres and Earth System/Key Laboratory of Marine Chemistry Theory and Technology, Ministry of Education, Ocean University of China, 238 Songling Road, Qingdao, 266100, P.R. China

^c^ Institute of Photochemistry and Photofunctional Materials, University of Shanghai for Science and Technology, 516 Jungong Road, Shanghai, 200093, P.R. China

* Email of corresponding authors: greta.patzke@chem.uzh.ch (Greta R. Patzke), liym@ouc.edu.cn (Yiming Li).

Table of Contents

[**Figure S1.** Scheme for the fabrication of CV-Ag-Ni_2_P/NF.	4](#_Toc228821177)

[**Figure S2.** LSV curves of Ag_n_Ni_1−n_/NF electrodes with different molar ratios of Ag:Ni in 1 M KOH for HER and OER, AOR in 1 M KOH and 0.5 M acrylamide without iR correction.	5](#_Toc228821178)

[**Figure S3.** SEM images of 500-CV-Ag-Ni_2_P/NF.	6](#_Toc228821179)

[**Figure S4.** EDX spectrum of CV-Ag-Ni_2_P/NF.	7](#_Toc228821180)

[**Figure S5.** XRD patterns of the different electrodes vs. reference Ni_2_P, Ni(OH)_2_, Ni and Ag..	8](#_Toc228821181)

[**Figure S6.** Calculated Ag valence state.	9](#_Toc228821182)

[**Figure S7.** KFPM images of Ni_2_P/NF, Ag-Ni_2_P/NF and CV-Ag-Ni_2_P/NF.	10](#_Toc228821183)

[**Figure S8.** LSV curves for the AOR of 1000-CV-Ni_2_P/NF and Ni_2_P/NF without iR correction.	11](#_Toc228821184)

[**Figure S9.** CV curves for the AOR of 1000-CV-Ni_2_P/NF before and after 20000 cycles of CV scanning test without iR correction.	12](#_Toc228821185)

[**Figure S10.** CV curves of the different electrodes with the scan rate ranging from 5 to 300 mV/s.	13](#_Toc228821186)

[**Figure S11.** Estimated ECSA values of the different electrodes with scan rates ranging from 5 to 300 mV/s.	14](#_Toc228821187)

[**Figure S12.** SEM images of CV-Ag-Ni_2_P/NF after HER tests.	15](#_Toc228821188)

[**Figure S13.** EDX elemental mapping images of 1000-CV-Ag−Ni_2_P, after HER tests and after AOR tests.	16](#_Toc228821189)

[**Figure S14.** SEM images of CV-Ag-Ni_2_P/NF after AOR tests.	17](#_Toc228821190)

[**Figure S15.** Standard curve of AA solutions; UV spectra of AA solutions before and after electrolysis.	18](#_Toc228821191)

[**Figure S16.** Degradation of AA by CV-Ag-Ni_2_P/NF || CV-Ag-Ni_2_P/NF electrode after 24 h.	19](#_Toc228821192)

[**Figure S17.** FT-IR spectra of AA solution before and after electrolysis, during different AOR reaction times.	20](#_Toc228821193)

[**Figure S18.** *In situ* Raman spectra of CV-Ag-Ni_2_P/NF electrode.	21](#_Toc228821194)

[**Figure S19.** ^13^C NMR spectrum (recorded at 100.61 MHz) of the sample AA, ethanol reference spectrum provided for comparison, and after AOR tests at different time intervals.	24](#_Toc228821195)

**Figure S20.** ^1^H NMR spectrum (recorded at 400.13 MHz) of the sample AA, ethanol reference spectrum provided for comparison, and after AOR tests at different time intervals.	……………………………………………………………………..27

[**Figure S21.** TOC removal during different AOR reaction times.	29](#_Toc228821197)

[**Figure S22.** HR-ESI-MS results for AA and after AOR tests.	31](#_Toc228821198)

[**Figure S23.** AA removal in the presence of nitrobenzene as a radical scavenger during AOR tests.	32](#_Toc228821199)

[**Figure S24.** Optimized adsorption models of Ni_2_P towards AOR in alkaline conditions..	33](#_Toc228821200)

[**Figure S25.** Optimized adsorption models of Ni_2_P towards HER in alkaline conditions..	34](#_Toc228821201)

[**Table S1.** BET surface areas of different electrode material samples.	35](#_Toc228821202)

[**Table S2.** Comparison of the HER performance of CV-Ag-Ni₂P/NF with representative Ni-based electrocatalysts.	36](#_Toc228821203)

[**Table S3.** Comparison of the CV-Ag-Ni_2_P/NF catalyst with other noble-metal-free catalysts for electrochemical oxidation of organic compounds.	37](#_Toc228821204)

[**Table S4.** Atomic percentage of elements of the catalysts derived from EDX analyses.	38](#_Toc228821205)

**Figure S1.** Scheme for the fabrication of CV-Ag-Ni_2_P/NF.

**Figure S2.** LSV curves of Ag_n_Ni_1−n_/NF electrodes with different molar ratios of Ag:Ni in
1 M KOH for HER and OER, AOR in 1 M KOH and 0.5 M acrylamide without iR correction.

**Figure S3.** SEM images of 500-CV-Ag-Ni_2_P/NF.

**Figure S4.** EDX spectrum of CV-Ag-Ni_2_P/NF.

**Figure S5.** XRD patterns of the different electrodes vs. reference Ni_2_P, Ni(OH)_2_, Ni and Ag.

**Figure S6.** Calculated Ag valence state.

**F****igure S7.** KFPM images of Ni_2_P/NF, Ag-Ni_2_P/NF and CV-Ag-Ni_2_P/NF.

**Figure S8.** LSV curves for the AOR of 1000-CV-Ni_2_P/NF and Ni_2_P/NF without iR correction.

**Figure S9.** CV curves for the AOR of 1000-CV-Ni_2_P/NF before and after 20000 cycles of CV scanning test without iR correction.

**Figure S10.** CV curves of the different electrodes with the scan rate ranging from 5 to 300 mV/s.

**Figure S11.** Estimated ECSA values of the different electrodes with scan rates ranging from 5 to 300 mV/s.

**Figure S12.** SEM images of CV-Ag-Ni_2_P/NF after HER tests.

**Figure S13.** EDX elemental mapping images of 1000-CV-Ag−Ni_2_P, after HER tests and after AOR tests.

**Figure S14.** SEM images of CV-Ag-Ni_2_P/NF after AOR tests.

**Figure S15.** Standard curve of AA solutions; UV spectra of AA solutions before and after electrolysis.

**Figure S16.** Degradation of AA by CV-Ag-Ni_2_P/NF || CV-Ag-Ni_2_P/NF electrode after 24 h.

**Figure S17.** FT-IR spectra of AA solution before and after electrolysis, during different AOR reaction times.

**Figure S18.** *In situ* Raman spectra of CV-Ag-Ni_2_P/NF electrode.

**Figure S19.** ^13^C NMR spectrum (recorded at 100.61 MHz) of AA, ethanol reference provided for comparison, and after AOR tests at different time intervals.

# Figure S20. ^1^H NMR spectrum (recorded at 400.13 MHz) of AA, ethanol reference provided for comparison, and after AOR tests at different time intervals.

# Ethanol quantification was performed by ¹H NMR using maleic acid (MA) as an internal standard. The concentration was determined from the integral ratio between the methyl proton signal of ethanol and the reference signal of MA. Signal assignments were validated by comparison with standard ethanol spectra. The ethanol concentration increased progressively with reaction time, reaching 0.0017 M after 2 h, 0.0084 M after 6 h, 0.0278 M after 24 h, and 0.0753 M after 36 h, respectively.

**Figure S21.** TOC removal during different AOR reaction times.

**Figure S22.** HR-ESI-MS results of AA, and after AOR tests.

**Figure S23.** AA removal in the presence of nitrobenzene as a radical scavenger during AOR tests.

**Figure S24.** Optimized adsorption models of Ni_2_P towards AOR in alkaline conditions.

**Figure S25.** Optimized adsorption models of Ni_2_P towards HER in alkaline conditions.

**Table S1.** BET surface areas of different electrode material samples.

| **Sample** | **BET surface area (m^2^/g)** |
| --- | --- |
| NF | 3.0 |
| Ag_0.5_Ni_0.5_(OH)_x_/NF | 5.5 |
| Ag-Ni_2_P/NF | 8.7 |
| CV-Ag-Ni_2_P/NF | 13.3 |

**Table S2.** Comparison of the HER performance of CV-Ag-Ni₂P/NF with representative Ni-based electrocatalysts.

| **Catalyst** | **Overpotential @10mA cm^-2^ (mV)** | **Overpotential @100 mA cm^-2^ (mV)** | **Tafel slope (mV dec^-1^)** | **electrolyte conditions** | **Ref.** |
| --- | --- | --- | --- | --- | --- |
| **CV-Ag-Ni_2_P/NF** | **104** | **288** | **48.2** | 1 M KOH | **This work** |
| S:CoP@NF | 109 | 185 | 54 | 1 M KOH | ^1^ |
| NiCoP/rGO | 59 | − | 50 | 0.5 M H_2_SO_4_ | ^2^ |
| Ni_2_P/Fe_2_P | 121 | − | 67 | 1 M KOH | ^3^ |
| NC/CuCo/CuCoO_x_  nanowire arrays | 112 | 224 | 55 | 1 M KOH | ^4^ |
| Mo-Ni_3_S_2_/Ni_x_P_y_/NF | 109 | 238-η_50_ | 68.4 | 1 M KOH | ^5^ |
| Ni-P-Ni/NF | 129 | 202 | 70 | 1 M KOH | ^6^ |
| Ni-ReSe_2_ | 109 | − | 81 | 1 M KOH | ^7^ |
| NiFe-LDHPOM/NF | 156 | 480 | 86 | 0.1 M KOH | ^8^ |
| 3D-NiCoP | 105 | − | 79 | 1 M KOH | ^9^ |
| Ni_5_P_4_/Ni_2_P/Fe_2_P-2 | 185 | 222 | 53.8 | 1 M KOH | ^10^ |
| Ce_0.10_-NiCoP | 96 | − | 94.75 | 1 M KOH | ^11^ |
| MoFeCoP | 285 | − | 83 | 1 M KOH | ^12^ |
| Ni_12_P_5_–Fe_2_P–NbP | − | 265 | 52 | 1 M KOH | ^13^ |
| RuCoNiP@α-Ni(OH)_2_ | − | 173.1 | 83 | 1 M KOH | ^14^ |

**Table S3.** Comparison of the CV-Ag-Ni_2_P/NF catalyst with other noble-metal-free catalysts for electrochemical oxidation of organic compounds.

| **Catalyst** | **electrolyte** | **Voltage**  **(V)** | ***j* (mA/cm^2^)** | **Ref.** |
| --- | --- | --- | --- | --- |
| **CV-Ag-Ni_2_P/NF** | **KOH + acrylamide** | **1.44** | **100** | **This work** |
| Ni_3_N/Mo_2_N | KOH + Urea | 1.54 | 100 | ^15^ |
| Ni_2_P/Ni\|\|Pt/C | KOH + Urea | 1.47 | 10 | ^16^ |
| CoMn/CoMn_2_O_4_ | KOH + Urea | 1.51 | 10 | ^17^ |
| NiFeO_x_-NF/NiFeN_x_-NF | KOH + glucose | 1.39 | 100 | ^18^ |
| MoO_2_-FeP@C | KOH + HMF | 1.486 | 10 | ^19^ |
| NiSe | KOH + α-nitrotoluenes | 1.36 | 10 | ^20^ |
| NiSe\|\|CoP | KOH + benzylamine | 1.49 | 20 | ^21^ |
| NiFe/CC | KOH +  5-hydroxymethylfurfural | 1.45 | 10 | ^22^ |
| Ni_3_N-V_2_O_3_ | KOH +  5-hydroxymethylfurfural | 1.44 | 10 | ^23^ |
| NiFe/Ni | KOH + furfural | 1.38 | 10 | ^24^ |
| F-doped NiCo_2_O_4_ | KOH +  5-hydroxymethylfurfural | 1.4 | 10 | ^25^ |

**Table S4.** Atomic percentage of elements of the catalysts derived from EDX analyses.

| **Sample** | **Atomic percentage of elements** | | | | | |
| --- | --- | --- | --- | --- | --- | --- |
|  | **Ni** | **Ag** | **P** | **O** | **C** | **Others** |
| CV-Ag-Ni_2_P/NF | 56.6 | 1.4 | 19.8 | 12.1 | 8.7 | 1.4 |
| After HER test | 41.1 | 2.4 | 4.0 | 31.7 | 15.8 | 5 |
| After AOR test | 36.5 | 6.6 | 3 | 33.8 | 16.7 | 3.4 |

Other elements：K, Au, Ca

**References**

(1) Anjum, M. A. R.; Okyay, M. S.; Kim, M.; Lee, M. H.; Park, N.; Lee, J. S. Bifunctional sulfur-doped cobalt phosphide electrocatalyst outperforms all-noble-metal electrocatalysts in alkaline electrolyzer for overall water splitting. *Nano Energy* **2018**, *53*, 286-295.

(2) Li, J.; Yan, M.; Zhou, X.; Huang, Z.-Q.; Xia, Z.; Chang, C.-R.; Ma, Y.; Qu, Y. Mechanistic Insights on Ternary Ni_2−x_Co_x_P for Hydrogen Evolution and Their Hybrids with Graphene as Highly Efficient and Robust Catalysts for Overall Water Splitting. *Adv. Funct. Mater.* **2016**, *26* (37), 6785-6796.

(3) Ge, Y.; Dong, P.; Craig, S. R.; Ajayan, P. M.; Ye, M.; Shen, J. Transforming Nickel Hydroxide into 3D Prussian Blue Analogue Array to Obtain Ni_2_P/Fe_2_P for Efficient Hydrogen Evolution Reaction. *Adv. Energy Mater.* **2018**, *8* (21), 1800484.

(4) Hou, J.; Sun, Y.; Wu, Y.; Cao, S.; Sun, L. Promoting Active Sites in Core–Shell Nanowire Array as Mott–Schottky Electrocatalysts for Efficient and Stable Overall Water Splitting. *Adv. Funct. Mater.* **2018**, *28* (4), 1704447.

(5) Luo, X.; Ji, P.; Wang, P.; Cheng, R.; Chen, D.; Lin, C.; Zhang, J.; He, J.; Shi, Z.; Li, N.; et al. Interface Engineering of Hierarchical Branched Mo-Doped Ni_3_S_2_/Ni_x_P_y_ Hollow Heterostructure Nanorods for Efficient Overall Water Splitting. *Adv. Energy Mater.* **2020**, *10* (17), 1903891.

(6) Zhang, J.; Zhang, Z.; Ji, Y.; Yang, J.; Fan, K.; Ma, X.; Wang, C.; Shu, R.; Chen, Y. Surface engineering induced hierarchical porous Ni_12_P_5_-Ni_2_P polymorphs catalyst for efficient wide pH hydrogen production. *Appl. Catal. B-Environ.* **2021**, *282*, 119609

(7) Kwon, I. S.; Kwak, I. H.; Ju, S.; Kang, S.; Han, S.; Park, Y. C.; Park, J.; Park, J. Adatom Doping of Transition Metals in ReSe_2_ Nanosheets for Enhanced Electrocatalytic Hydrogen Evolution Reaction. *ACS Nano* **2020**, *14* (9), 12184-12194.

(8) Li, C.; Zhang, Z.; Liu, R. In Situ Growth of 3D NiFe LDH-POM Micro-Flowers on Nickel Foam for Overall Water Splitting. *Small* **2020**, *16* (46), 2003777.

(9) Ma, B.; Yang, Z.; Chen, Y.; Yuan, Z. Nickel cobalt phosphide with three-dimensional nanostructure as a highly efficient electrocatalyst for hydrogen evolution reaction in both acidic and alkaline electrolytes. *Nano Res.* **2019**, *12* (2), 375-380.

(10) Sun, S.; Zhang, C.; Ran, M.; Zheng, Y.; Li, C.; Jiang, Y.; Yan, X. Fe-doped promotes phosphorization and dispersibility of Ni catalysts for efficient and stable HER and OER. *Int. J. Hydrogen Energy* **2024**, *63*, 133-141.

(11) Zhang, M.; Xu, H.; Yang, H.; Shang, X.; Yuan, M.; Fu, Y.; Xiao, Y.; Wang, S.; Wang, X.; Jia, B.; et al. Electronic Modulation of Nickel Cobalt Phosphide Nanosheets by Ce Doping for Efficient Overall Water Splitting. *Small* **2025**, *21* (32), 2504837.

(12) Streckova, M.; Fedorockova, A.; Guboova, A.; Sučik, G.; Girman, V.; Hussain, A.; Vorochta, M.; Strečka, J.; Bystron, T. Synthesis of Hollow Spherical Phosphide Catalysts with Industrial-Scale Potential for Alkaline Hydrogen Evolution Reaction. *ACS Appl. Mater. Interf.* **2025**, *17* (39), 54749-54760.

(13) Wen, S.; Chen, G.; Chen, W.; Li, M.; Ouyang, B.; Wang, X.; Chen, D.; Gong, T.; Zhang, X.; Huang, J.; et al. Nb-doped layered FeNi phosphide nanosheets for highly efficient overall water splitting under high current densities. *J. Mater. Chem. A* **2021**, *9* (15), 9918-9926.

(14) Lian, W.; Chen, Z.; Mo, H.; Zhu, Q.; Wu, J.; Zhang, X.; Song, S.; Jia, F. Deciphering the surface electrochemical reconstruction of ruthenium-cobalt-nickel phosphide for efficient high-current hydrogen evolution and overall water splitting. *J. Colloid Interf. Sci.* **2025**, *683*, 291-300.

(15) Wang, T.; Miao, L.; Zheng, S.; Qin, H.; Cao, X.; Yang, L.; Jiao, L. Interfacial Engineering of Ni_3_N/Mo_2_N Heterojunctions for Urea-Assisted Hydrogen Evolution Reaction. *ACS Catal.* **2023**, *13* (7), 4091-4100.

(16) Li, Q.; Li, X.; Gu, J.; Li, Y.; Tian, Z.; Pang, H. Porous rod-like Ni_2_P/Ni assemblies for enhanced urea electrooxidation. *Nano Res.* **2021**, *14* (5), 1405-1412.

(17) Wang, C.; Lu, H.; Mao, Z.; Yan, C.; Shen, G.; Wang, X. Bimetal Schottky Heterojunction Boosting Energy-Saving Hydrogen Production from Alkaline Water via Urea Electrocatalysis. *Adv. Funct. Mater.* **2020**, *30* (21), 2000556.

(18) Liu, W.; Xu, Z.; Zhao, D.; Pan, X.; Li, H.; Hu, X.; Fan, Z.; Wang, W.; Zhao, G.; Jin, S.; et al. Efficient electrochemical production of glucaric acid and H_2_ via glucose electrolysis. *Nat. Commun.* **2020**, *11* (1), 265.

(19) Yang, G.; Jiao, Y.; Yan, H.; Xie, Y.; Wu, A.; Dong, X.; Guo, D.; Tian, C.; Fu, H. Interfacial Engineering of MoO_2_-FeP Heterojunction for Highly Efficient Hydrogen Evolution Coupled with Biomass Electrooxidation. *Adv. Mater.* **2020**, *32* (17), 2000455.

(20) Chong, X.; Liu, C.; Wang, C.; Yang, R.; Zhang, B. Integrating Hydrogen Production and Transfer Hydrogenation with Selenite Promoted Electrooxidation of α-Nitrotoluenes to E-Nitroethenes. *Angew. Chem. Int. Ed.* **2021**, *60* (40), 22010-22016.

(21) Huang, Y.; Chong, X.; Liu, C.; Liang, Y.; Zhang, B. Boosting Hydrogen Production by Anodic Oxidation of Primary Amines over a NiSe Nanorod Electrode. *Angew. Chem. Int. Ed.* **2018**, *57* (40), 13163-13166.

(22) Liu, H.; Lee, T.; Chen, Y.; Cochran, E. W.; Li, W. Paired and Tandem Electrochemical Conversion of 5-(Hydroxymethyl)furfural Using Membrane-Electrode Assembly-Based Electrolytic Systems. *ChemElectroChem* **2021**, *8* (15), 2817-2824.

(23) Liang, S.; Pan, L.; Thomas, T.; Zhu, B.; Chen, C.; Zhang, J.; Shen, H.; Liu, J.; Yang, M. Ni_3_N-V_2_O_3_ enables highly efficient 5-(Hydroxymethyl) furfural oxidation enabling membrane free hydrogen production. *Chem. Eng. J.* **2021**, *415*, 128864.

(24) Wang, W.; Yu, X.; He, H.; Wang, Y.; Li, Y.; Deng, L.; Liu, Y.-N. Electrochemical reconstitution of Prussian blue analogue for coupling furfural electro-oxidation with photo-assisted hydrogen evolution reaction. *Chem. Eng. J.* **2023**, *465*, 142865.

(25) Yang, S.; Xiang, X.; He, Z.; Zhong, W.; Jia, C.; Gong, Z.; Zhang, N.; Zhao, S.; Chen, Y. Anionic defects engineering of NiCo_2_O_4_ for 5-hydroxymethylfurfural electrooxidation. *Chem. Eng. J.* **2023**, *457*, 141344.
